# Supplementary material for: Influenza-associated hospitalisation, vaccine uptake and socioeconomic deprivation in an English city region: an ecological study
Source: BMJ Open. 2018 Dec 19;8(12):e023275. doi: 10.1136/bmjopen-2018-023275 (PMC6303586; doi:10.1136/bmjopen-2018-023275)
Supplement: Supplementary file 1 [file bmjopen-2018-023275supp001.pdf]

S1-Table 1. ICD-10 codes used to identify comorbidities adapted from Cromer et al. 2014

Condition	ICD-10 codes used
**Chronic respiratory disease**	J4, J6, J7, J8, Q30, J31, Q32, Q33Q34, Q35, Q36, Q37
**Chronic heart disease**	I05, I06, I07, I08, I09, I11, I12, I13, I20, I21, I22, I25, I27, I28, I3, I40, I41, I42, I43, I44, I45, I47, I48, I49, I5, I6, Q2
**Chronic kidney disease**	N0, N11, N12, N14, N15, N16, N18, N19, N25, Q60, Q61
**Chronic liver disease**	K7, P78.8, Q44, B18
**Chronic neurological disease**	G1, G2, G3, G4, G5, G6, G7, G8, G9
**Diabetes**	E10, E11, E12, E13, E14, E24, G59.0, G63.2, G73.0, G99.0, N08.3, O24, P70.0, P70.1, P70.2
**Immunosuppression**	All C-codes, D37, D38, D39, D4, B20, B21, B22, B23, B24, Z94, Z85, Z94.8, D56.1, D57.8, D57.0, D57.D61, D70, D71, D72, D73, D76, D80, D81, D82, D83, D84, K90.0
**Other including: asplenia or dysfunction of the spleen, cochlear implants and cerebrospinal fluid leaks**	D73, D56.1, D57.8, D57.0, D57.1, K90.0, Z96.1, G96.0

S1-Table 2. Demographics of Merseyside residents admitted to hospital with IAI (ICD-10: J09, J10, J128, J129, J11, J22, J069) by financial year, 2004/05 to 2015/16 (N=89,058)

	04/05 (N=7457)		05/06 (N=8018)		06/07 (N=9248)		07/08 (N=8175)		08/09 (N=8269)		09/10 (N=7559)		10/11 (N=7640)		11/12 (N=5231)		12/13 (N=6399)		13/14 (N=5883)		14/15 (N=6985)		15/16 (N=8194)	
Sex	**n**	**%**	**n**	**%**	**n**	**%**	**n**	**%**	**n**	**%**	**n**	**%**	**n**	**%**	**n**	**%**	**n**	**%**	**n**	**%**	**n**	**%**	**n**	**%**
Female	3900	52	4173	52	4572	49	4069	50	4171	50	3759	50	3777	49	2633	50	3267	51	3048	52	3730	53	4190	51
Male	3556	48	3845	48	4675	51	4106	50	4098	50	3800	50	3863	51	2598	50	3132	49	2835	48	3255	47	4004	49
Unknown	1	0	0	0	1	0	0	0	0	0	0	0	0	0	0	0	0	0	0	0	0	0	0	0
**Age group**																								
<24m	1281	17	1544	19	2583	28	2089	26	2310	28	2376	31	2235	29	1250	24	1441	23	1315	22	1425	20	1572	19
24-59m	510	7	597	7	1057	11	857	10	908	11	954	13	1013	13	589	11	692	11	643	11	680	10	873	11
5-14y	309	4	449	6	565	6	412	5	492	6	575	8	523	7	313	6	383	6	277	5	340	5	490	6
15-39y	752	10	825	10	761	8	844	10	708	9	628	8	785	10	463	9	581	9	605	10	797	11	1131	14
40-64y	1363	18	1376	17	1290	14	1312	16	1186	14	1049	14	1057	14	829	16	1104	17	1053	18	1277	18	1473	18
65+	3242	43	3227	40	2992	32	2661	33	2665	32	1977	26	2027	27	1787	34	2198	34	1990	34	2466	35	2655	32
**National IMD quintile**																								
5 (least deprived)	410	5	468	6	511	6	449	5	497	6	400	5	452	6	348	7	427	7	384	7	514	7	594	7
4	855	11	946	12	1005	11	959	12	914	11	831	11	862	11	654	13	837	13	764	13	880	13	1060	13
3	983	13	1107	14	1255	14	1038	13	1107	13	986	13	1007	13	762	15	996	16	856	15	998	14	1094	13
2	1151	15	1250	16	1445	16	1193	15	1292	16	1096	14	1096	14	818	16	1013	16	940	16	1149	16	1397	17
1 (most deprived)	4058	54	4247	53	5032	54	4536	55	4459	54	4246	56	4223	55	2649	51	3126	49	2939	50	3444	49	4049	49
**Comorbidity**																								
**No**	3617	49	4109	51	5442	59	4709	58	4528	55	4357	58	4360	57	2553	49	3046	48	2757	47	3073	44	3795	46
**Yes**	3840	51	3909	49	3806	41	3466	42	3741	45	3202	42	3280	43	2678	51	3353	52	3126	53	3912	56	4399	54

IMD: Index of Multiple Deprivation; m: months; y: years

S1-Table 3. Interaction between socioeconomic deprivation (IMD quintile) and age group on the rate of ILI hospitalisation in Merseyside, UK

	Mean yearly rate per 1,000 population	IRR	Full model with interaction: IRR	Standard error	P value
**Main effects**					
National IMD quintile					
5 (least deprived)	3.46	Reference	Reference		
4	3.98	1.13 (1.05-1.21)	1.00 (0.86-1.16)	0.077	0.984
3	4.26	1.18 (1.10-1.27)	0.99 (0.85-1.15)	0.076	0.864
2	4.82	1.37 (1.27-1.47)	1.04 (0.90-1.21)	0.076	0.581
1 (most deprived)	5.84	1.61 (1.50-1.72)	1.1 (0.95-1.27)	0.074	0.19
Age group					
<24m (reference)	51.3	Reference	Reference		
24-59m	15.4	0.30 (0.28-0.32)	0.29 (0.24-0.34)	0.084	<0.001
5-14y	2.49	0.05 (0.04-0.05)	0.05 (0.04-0.05)	0.090	<0.001
15-39y	1.50	0.03 (0.02-0.03)	0.02 (0.02-0.02)	0.091	<0.001
40-64y	2.43	0.04 (0.04-0.04)	0.03 (0.02-0.03)	0.082	<0.001
65+	9.66	0.18 (0.16-0.19	0.13 (0.11-0.15)	0.077	<0.001
Year	4.94	0.99 (0.98-1.00)	0.99 (0.98-1.00)	0.003	<0.001
Sex					
Male	5.01	Reference	Reference		
Female	4.88	0.98 (0.94-1.02)	0.98 (0.94-1.02)	0.020	0.263
**Interaction effects (IMD quintile interacted with age group)**					
IMD quintile 5 * <24m			reference		
IMD quintile 4 * 24-59m	-	-	1.05 (0.84-1.31)	0.114	0.663
IMD quintile 3 * 24-59m	-	-	1.06 (0.85-1.32)	0.113	0.628
IMD quintile 2* 24-59m	-	-	1.10 (0.89-1.38)	0.113	0.38
IMD quintile 1 * 24-59m	-	-	1.04 (0.84-1.29)	0.109	0.736
IMD quintile 4 * 5-14y	-	-	1.08 (0.85-1.37)	0.121	0.524
IMD quintile 3 * 5-14y	-	-	0.95 (0.75-1.21)	0.121	0.7
IMD quintile 2 * 5-14y	-	-	1.17 (0.93-1.48)	0.119	0.187
IMD quintile 1 * 5-14y	-	-	1.11 (0.88-1.39)	0.114	0.375
IMD quintile 4 * 15-39y	-	-	1.17 (0.92-1.48)	0.121	0.205
IMD quintile 3 * 15-39y	-	-	1.33 (1.06-1.68)	0.119	0.016
IMD quintile 2 * 15-39y	-	-	1.34 (1.06-1.69)	0.118	0.014
IMD quintile 1 * 15-39y	-	-	1.88 (1.50-2.35)	0.115	<0.001
IMD quintile 4 * 40-64y	-	-	1.25 (1.00-1.55)	0.112	0.05
IMD quintile 3 * 40-64y	-	-	1.57 (1.26-1.95)	0.111	<0.001
IMD quintile 2 * 40-64y	-	-	1.79 (1.44-2.22)	0.110	<0.001
IMD quintile 1 * 40-64y	-	-	2.39 (1.94-2.95)	0.108	<0.001
IMD quintile 4 * 65+	-	-	1.26 (1.03-1.56)	0.106	0.026
IMD quintile 3 * 65+	-	-	1.39 (1.13-1.71)	0.105	0.002
IMD quintile 2 * 65+	-	-	1.65 (1.34-2.02)	0.105	<0.001
IMD quintile 1 * 65+	-	-	1.75 (1.43-2.14)	0.103	<0.001

IMD: Index of Multiple deprivation; IRR: Incidence rate ratio; m: months; y: years
