# Supplementary material for: Influenza-associated hospitalisation, vaccine uptake and socioeconomic deprivation in an English city region: an ecological study
Source: BMJ Open. 2018 Dec 19;8(12):e023275. doi: 10.1136/bmjopen-2018-023275 (PMC6303586; doi:10.1136/bmjopen-2018-023275)
Supplement: Author's manuscript [file bmjopen-2018-023275.draft_revisions.pdf]

BMJ Open is committed to open peer review. As part of this commitment we make the peer review history of every article we publish publicly available.

When an article is published we post the peer reviewers' comments and the authors' responses online. We also post the versions of the paper that were used during peer review. These are the versions that the peer review comments apply to.

The versions of the paper that follow are the versions that were submitted during the peer review process. They are not the versions of record or the final published versions. They should not be cited or distributed as the published version of this manuscript.

BMJ Open is an open access journal and the full, final, typeset and author-corrected version of record of the manuscript is available on our site with no access controls, subscription charges or pay-per-view fees (http://bmjopen.bmj.com).

If you have any questions on BMJ Open's open peer review process please email info.bmjopen@bmj.com

BMJ Open

Influenza associated hospitalisation, vaccine uptake and socioeconomic deprivation in an English city region: an ecological study

Journal:	*BMJ Open*
Manuscript ID	bmjopen-2018-023275
Article Type:	Research
Date Submitted by the Author:	29-Mar-2018

Complete List of Authors:	Hungerford, Daniel; University of Liverpool, Centre for Global Vaccine Research, Institute of Infection and Global Health; Public Health England, Field Epidemiology Service, Health Protection<br>Ibarz-Pavon, Ana; University of Liverpool, Centre for Global Vaccine Research, Institute of Infection and Global Health<br>Cleary, Paul; Public Health England, Field Epidemiology Service<br>French, Neil; The Centre for Global Vaccine Research, Institute of Infection & Global Health; Malawi-Liverpool-Wellcome Trust Clinical Research Programme, College of Medicine, University of Malawi

Keywords:	Epidemiology < INFECTIOUS DISEASES, Public health < INFECTIOUS DISEASES, Inequalities, Influenza, Vaccination, ecological

SCHOLARONE™ Manuscripts

Influenza associated hospitalisation, vaccine uptake and socioeconomic deprivation in an English city region: an ecological study

Daniel Hungerford[1,2]*, Ana Ibarz-Pavon[1], Paul Cleary[2], Neil French[1]

1. The Centre for Global Vaccine Research, Institute of Infection and Global Health, University of Liverpool, Liverpool, UK

2. Field Epidemiology Service, National Infection Service, Public Health England, Liverpool, UK

Word count=3588

*Correspondence: d.hungerford@liverpool.ac.uk

Short title: Hospitalised influenza associated illness, vaccine uptake and deprivation in the UK

For peer review only

For peer review only - http://bmjopen.bmj.com/site/about/guidelines.xhtml

Abstract

Objectives: Every year, influenza poses a significant burden on the National Health Service in England. Influenza vaccination is an effective measure to prevent severe disease, hence, maximising vaccine coverage in the most vulnerable is a priority. We aimed to identify the extent to which socioeconomic status is associated with influenza-associated illness (IAI) and influenza vaccine coverage.

Design: Retrospective observational study using hospital episode statistics.

Setting: Merseyside, North-West of England, including the city of Liverpool.

Participants: Residents of Merseyside hospitalised with IAI between April 2004 and March 2016, and Merseyside general practice registered patients eligible for influenza vaccination in 2014/15 and 2015/16 influenza seasons.

Exposures: Socioeconomic deprivation based on lower super output area English Indices of Deprivation scores

Primary and secondary outcome measures: Incidence and risk of IAI hospitalisation, and vaccine uptake

Results: There were 89,058 hospitalisations related to IAI among Merseyside residents (mean yearly rate = 4.9 per 1,000 population). Hospitalisations for IAI were more frequent in the most socioeconomically deprived areas compared to the least deprived in adults aged 15-39 years (incidence rate ratio [IRR]=2.08: 95% CI; 1.76-2.45; p<0.001), 60-64 years (IRR=2.65: 95% CI; 2.35-2.99; p<0.001) and 65+ years (IRR=1.90: 95% CI; 1.73-2.10; p<0.001); whereas rates in children were more homogeneous across deprivation strata. Vaccine uptake was lower than the nationally-set targets in most neighbourhoods. The odds of vaccine uptake were 30% lower (odds ratio [OR]= 0.70: 95% CI; 0.66-0.74; p<0.001) and 10% lower (OR= 0.90: 95% CI; 0.88-0.92; p<0.001) in the most deprived IMD quintile compared to the least deprived among children aged 24-59 months and 65+

For peer review only - http://bmjopen.bmj.com/site/about/guidelines.xhtml

year olds, respectively.

Conclusions: Higher rates of IAI hospitalisations and lower vaccine uptake in the most socioeconomically deprived populations suggest that health promotion policies and interventions that target these populations should be a priority.

Strengths and limitations of study methodology

- The study uses routine public health data sources and a simple repeatable methodology which can be applied to continue to monitor changes in incidence and vaccine uptake in local areas, and potentially evaluate targeted population-level interventions.
- We were unable to evaluate population-level impact of influenza vaccines because of non-specific endpoints and seasonal disease variation over the study period.
- While an association with socioeconomic deprivation was demonstrated for incidence of hospitalisation and uptake independently, difficulties linking data recorded through several incompatible databases prevented integration and development of more complex models.
- Further research is needed to understand the attitudes and beliefs toward the flu vaccine influence its acceptability and uptake among the population

For peer review only - http://bmjopen.bmj.com/site/about/guidelines.xhtml

Background

Influenza is a highly infectious disease associated with high morbidity and mortality rates among vulnerable populations worldwide. Although influenza imposes a considerable burden on the National Health Service (NHS) in the United Kingdom (UK), estimating the true burden of disease is difficult due to the non-specific symptomatic presentation of disease and lack of laboratory confirmation. In England and Wales, influenza is thought to account for almost 900,000 GP consultations, 25,000 hospitalisations and 20,000 deaths every year [1]. However, several studies attempting to estimate the true burden of disease using different approaches and statistical modelling suggest that burden of illness attributable to influenza in the UK is likely to be underestimated by current surveillance systems [1–5]. Children <5 years of age and those aged 65+ years and individuals with underlying comorbidities are known to be at higher risk of complicated influenza [2,6].Socioeconomic deprivation has also been associated with higher influenza-related mortality and hospitalisations [7–9].

Vaccination is the most effective control measure against complications of seasonal influenza. In the UK, the vaccine is offered to those aged 65 and over, and to individuals at risk of complicated influenza under 65 years of age. In 2013, the Department of Health (DH) expanded vaccination to children with an age-phased rollout of an intranasal Live Attenuated Influenza Vaccine (LAIV) programme that at the time of writing, includes all children aged 2-8 years, and will eventually target all those up to 17 years of age [10]. Children aged 2-4 years receive the vaccine at their general practice (GP) while those aged 5-8 are vaccinated at school. During the 2016/17 influenza season, vaccine uptake in England was 70.5% among those aged 65+ years, 36.9% among children aged 2-4 years, 48.6% in risk groups aged 6 months to 64 years and 55.4% among children aged 5-8 years in school years 1 to 3 [11,12]. However, there is variation in uptake between GP practices and few practices achieve the 40% LAIV uptake target in 2-4 year olds [13]. There is an inverse association between deprivation and uptake of routine childhood vaccines (*e.g.* vaccines for measles, mumps and rubella, human papillomavirus, influenza) [14–16]. Therefore, optimizing vaccine delivery and

For peer review only - http://bmjopen.bmj.com/site/about/guidelines.xhtml

identifying key target areas is necessary to ensure the protection of those most vulnerable, and to maximize cost-effectiveness. Simulation studies supporting the decision to include LAIV in the vaccination schedule indicated that increasing vaccine uptake in children from 30% to 60% can markedly increase both direct and indirect protective effects of the vaccine [1,17,18]. However, these studies assume uniform vaccine coverage and fail to account for the substantial variation in disease risk and vaccine uptake observed across local areas with different socioeconomic status [15]. Given that comorbidities that increase the risk of adverse outcomes from influenza are substantially more prevalent among the most deprived populations [19,20], it would be expected that ensuring vaccine delivery to the most vulnerable groups would increase cost-effectiveness. Understanding how vaccine uptake varies across local areas with differing socioeconomic status, and correlating this to disease burden and use of healthcare resources, will provide evidence to inform immunisation policy and assist in developing targeted vaccine interventions to mitigate this effect. The aim of this study was to describe the burden of severe influenza associated illness (IAI) in secondary care across local communities in a defined population in relation to small area level socioeconomic deprivation and vaccine uptake levels. This information will help to identify priority populations for public health interventions to increase vaccine uptake and to reduce the health and economic impact of influenza.

Methods

Population and Setting

The study population was the metropolitan area of Merseyside, North-West England, defined in this study as the local authority areas of Halton, Knowsley, Liverpool, Sefton, St. Helens, and Wirral. Merseyside has an estimated resident population of 1.5 million [21], and is a diverse area that contains some of the most socioeconomically deprived neighbourhoods in England as well as some of the most affluent [22].

Data sources

Influenza vaccine uptake

For peer review only - http://bmjopen.bmj.com/site/about/guidelines.xhtml

Influenza vaccine uptake data for seasons 2014/15 and 2015/16 were obtained from ImmForm, the system used by the DH, the NHS, and Public Health England (PHE) to record general practice vaccination uptake [21]. We extracted aggregated information on vaccine uptake for all groups eligible for vaccination from 288 GP practices in Merseyside. Data included the number of eligible patients and the number of people receiving the vaccine for each eligible group.

Hospitalisations for influenza-associated illness

Hospitalisation data were extracted from the national Hospital Episode Statistics (HES) database, a records-based system maintained by NHS digital, which contains information on all hospital admissions, outpatient appointments, and A&E attendances covering all NHS trusts in England.

We extracted anonymised aggregated records of all patients residing in Merseyside admitted as an emergency to a hospital in England with a primary or subsequent diagnosis of acute respiratory illness identified between April 2004 and March 2016. We used the International Statistical Classification of Diseases and Related Health Problems (ICD-10) codes (ICD-10 codes J0*, J1*, J2*, J3*) to identify acute respiratory infection [2]. We further used the ICD10-coding to identify confirmed influenza, IAI, and comorbidities. Confirmed influenza was identified by codes J09 and J10. IAI codes were selected using the following procedure and criteria:

- Literature search of peer-reviewed publications [2,23,24]
- Identification of ICD-10 codes used by the NHS trusts as a primary diagnostic of respiratory diseases during the peak influenza season (December to March). We calculated means of daily usage during the peak influenza compared to the rest of year and excluded codes which had a daily usage ratio of less than three during the peak influenza season compared to the rest of the year [25].
- Excluding specific ICD-10 codes which were identifying non-influenza related illness, such as, for example, J211-RSV pneumonia, J123-Human Metapneumovirus pneumonia and J301-Allergic rhinitis due to pollen.

For peer review only

For peer review only - http://bmjopen.bmj.com/site/about/guidelines.xhtml

- Excluding ICD-10 codes (J218 and J219) commonly used for Respiratory Syncytial Virus (RSV). We identified these codes through a prior study at a major acute care hospital in Merseyside by cross-checking patients with laboratory confirmed RSV with their ICD-10 primary diagnosis code on discharge (unpublished data).

Final selected ICD-10 codes for IAI were as follows: J06.9 (unspecified acute upper respiratory tract infection), J09 (influenza due to certain identified influenza virus), J10 (Influenza due to other identified influenza virus), J11 (Influenza, virus not identified), J12.8 (Other viral pneumonia), J12.9 (Viral pneumonia, unspecified), J22 (unspecified acute lower respiratory tract infection). ICD-10 codes used to identify comorbidities are listed in supplementary S1-Table 1 and were identified using the PHE Flu Plan 2016/17 and Cromer *et al.* 2014 [2,26].

Area of residence, population denominators and socioeconomic deprivation

The HES data set includes a code for neighbourhood areas of residence, known as Lower Super Output Areas (LSOAs). There are 32,482 LSOAs in England, each containing approximately 1,500 persons [21]. These statistical boundaries were constructed after the 2011 census, and have an associated measure of socioeconomic deprivation: the English Indices of Deprivation 2015, of which the Index of Multiple Deprivation (IMD) is the most commonly used. The IMD is a composite measure of social deprivation based on seven weighted domains: income; employment; health and disability; education skills and training; barriers to housing and other services; crime; and living environment [22]. Population denominators at LSOA and middle super output area (MSOA) were accessed for 2004 to 2015 by single year of age and sex and aggregated into the appropriate age categories referred to below. This information is published by the Office for National Statistics (ONS) and held by PHE [21].

Statistical analysis

Hospitalisation data from HES were recoded, reshaped, and aggregated by age, sex, and time before

For peer review only - http://bmjopen.bmj.com/site/about/guidelines.xhtml

merging to other data sources. Age categories were constructed based on age at hospitalisation: <24m; 24-59m; 5-14y; 15-39y; 40-64y; 65+.

To analyse the spatial and temporal changes in IAI hospitalisations, we constructed age and sex adjusted standardised incidence ratios (SIRs) for Merseyside at the MSOA level (approximately 5,000-12,000 resident population) to avoid small numbers. SIRs that represent the relative risk of IAI hospitalisation in an MSOA were compared to the Merseyside average. To calculate SIRs, we first calculated the expected number of hospitalisations in each $MSOA_{ijk}$ by calculating overall Merseyside rates for each combination of age $_j$ and sex $_k$ and multiplying these by corresponding MSOA population denominators, as exampled in the following equation:

$$Expected\ number_{ijk} = \frac{MSOA\ population_{jk}}{Total\ population_{jk}} \times Total\ number\ of\ hospitalisations_{jk}$$

Expected numbers are summed for each MSOA and SIRs are calculated by the ratio of the observed to expected numbers in each MSOA, as shown below:

$$SIR_i = \frac{Observed\ number\ of\ hospitalisations_i}{Expected\ number\ of\ hospitalisations_i}$$

We then produced choropleth maps of MSOA SIR for four three-year pooled time periods for the area of Merseyside.

For analyses focusing on associations between socioeconomic deprivation and hospitalisation or uptake, we first constructed socioeconomic deprivation groups for IMD using quintiles of their score for LSOAs in England. In 2015, 45% of the population in Merseyside lived in the most deprived quintile [22]. Association between socioeconomic status and IAI hospitalisations were assessed by fitting a negative binomial regression model (due to over-dispersion) offset by resident population, adjusting for age group, sex and year-by-year variability in influenza incidence by including fiscal year. To examine the association between age group and deprivation an interaction term was also included. We also stratified by age group and incidence rate ratios (IRR) were calculated along with

For peer review only - http://bmjopen.bmj.com/site/about/guidelines.xhtml

95% confidence intervals.

To estimate the association between socioeconomic deprivation and influenza vaccine uptake, uptake data for each LSOA were extracted at GP practice level. We used the LSOA level GP registered population, weighting vaccine uptake by proportion of population served by each GP practice, to create synthetic estimates of vaccine uptake at LSOA level for the 2014/15 and 2015/16 seasons. Vaccine uptake levels were classified into three categories in accordance with the national targets set by NHS England, DH and PHE [26]:

- 24-59 month olds (target: 40%)
- 65+ year olds (target: 75%)
- At risk groups under 65 years of age (target: 55%)

The association between socioeconomic deprivation and vaccine uptake was assessed using fractional logit regression and robust standard errors [27]. The dependent variable was vaccine uptake, included as a proportion, and the independent variable was IMD quintile. The model was adjusted for fiscal year (April to March) and, because the unit of analysis was LSOA, the model was weighted using LSOA population size. Three separate models were constructed for vaccine uptake in 24-59 month olds, 65+ year olds, and at-risk groups under 65 years of age. The models enabled the calculation of odds ratios (ORs) and robust 95% confidence intervals.

Demographic characteristics were compared between those with and without clinical risk factors for severe IAI. Continuous variables were tested by Student's t-test, or Wilcoxon rank-sum test if not normally distributed, and χ^2-test or Fisher's exact test were used for categorical variables.

All data processing and statistical analyses were conducted using R v. 3.2.3 [28].

For peer review only - http://bmjopen.bmj.com/site/about/guidelines.xhtml

Results

Hospitalisations in Merseyside

Between April 2004 and March 2016, there were 89,058 hospitalisation events coded as influenza or IAI in Merseyside (mean *per* year: 7,421.5; 4.9 per 1,000 population), of which 43,761 (49.1%) occurred among male patients (supplementary S1-Table 2). The highest number of hospitalisations occurred in the 2006/07 season (n=9,247; 6.2 per 1,000 population), and the lowest in 2011/12 (5,231; 3.5 per 1,000 population). Hospitalisations showed a clear seasonal pattern, peaking in December and January (Figure 1). Most hospitalisations (n=29,877; 33.5%) corresponded to patients aged 65 years and over, or children under 24 months of age (n=21,420; 24%). Over the study period, the mean population rate of hospitalisation was highest in children aged under 24 months of age (51.3 per 1,000) and 24-59 months of age (15.4 per 1,000).

During the 2009/10 influenza season, when the H1N1 pandemic occurred, the proportion of hospitalisations corresponding to patients 65+ year olds was at its lowest (Figure 1; supplementary S1-Table 2), reflecting the shift towards younger adults observed during the H1N1 pandemic. Forty-eight percent (n=42,712) of hospitalisation had an ICD-10 clinical code that indicated a comorbidity. The two most common comorbidities were heart disease (n=19,293; 45%) and chronic respiratory conditions (n=15,755; 37%). The proportion of hospitalisations with comorbidities for complicated influenza increased with age, and was slightly greater in males (n=22,444; 53%).

Association between hospitalisation rates and socioeconomic deprivation

Over half of the hospitalisations (n=47,008; 53%) occurred among those living in the most socioeconomically deprived quintile (Table 1). Overall, the proportion of hospitalisations for IAI with a comorbidity was lower in the most deprived quintile (47%: n=22167/47008; p=0.01) compared to the least deprived (49%: n=2672/5454) (Table 1). In respect to age group, a similar proportion of children aged <24 months (range: 6-7%) and adults 65+ years of age (range 79-81%) had comorbidities across the deprivation strata (Table 1). However, among children aged 5-14 years and

For peer review only - http://bmjopen.bmj.com/site/about/guidelines.xhtml

among 15-39 year olds and 40-64 years olds the proportion of IAI hospitalisations that had a comorbidity was highest in the most deprived quintiles (Table 1). Below the age of 5 years there was little difference in hospitalisation rates between the deprivation quintiles (Figure 2); for example, the mean rate of IAI hospitalisations over the study period was 26.5 per 1,000 population in the most deprived quintile and 31.4 per 1,000 population in the least deprived. For children aged 5-14 years the risk of IAI hospitalisation was 1.2 times among those from the most deprived compare to the least deprived (IRR=1.23: 95% CI; 1.04-1.47; p=0.019). In those aged 15-39 years the risk of hospitalisation was over 2 times higher (IRR=2.08: 95% CI; 1.76-2.45; p<0.001) among residents in the most deprived areas in comparison to those in the least deprived quintile; in adults aged 40-64 years it was 2.6 times higher (IRR=2.65: 95% CI; 2.35-2.99; p<0.001) and 1.9 times higher in 65+ year olds (IRR=1.90: 95% CI; 1.73-2.10; p<0.001). When including an interaction term for the association between IMD quintile and age group, socioeconomic deprivation was strongly associated with an increased rate of hospitalisation in the 15-39, 40-64 and 65+ year age group. Comparing the least deprived IMD quintile with the most deprived and 40-64 year olds with <24 month olds, risk of hospitalisation was nearly 2.5 times higher (IRR= 2.39: 95% CI 1.94-2.95; p<0.001) (supplementary S1-Table 3). Spatially age and sex-standardised Incidence ratios (SIRs) for IAI hospitalisations also show a visual association with deprivation over time, with higher rates being consistently observed in the most deprived areas (Figure 3).

For peer review only

Table 1. Population of Merseyside admitted to hospital with IAI by national IMD quintile between 2004/05 and 2015/16 (N=89058).

	National Index of Multiple Derivation quintile										Total	
	5 (least deprived) (N=5454)		4 (N=10567)		3 (N=12189)		2 (N=13840)		1 (most deprived) (N=47008)			
**Sex**	n	%	n	%	n	%	n	%	n	%	n	%
Female	2773	51	5394	51	6192	51	7121	51	23809	51	45289	51
Male	2681	49	5173	49	5997	49	6719	49	23197	49	43767	49
Unknown	0	0	0	0	0	0	0	0	2	0	2	0
**Age group**												
<24m	1205	22	2344	22	2898	24	3195	23	11779	25	21421	24
24-59m	607	11	1115	11	1290	11	1444	10	4917	10	9373	11
5-14y	385	7	679	6	634	5	801	6	2629	6	5128	6
15-39y	343	6	769	7	1079	9	1290	9	5399	11	8880	10
40-64y	742	14	1517	14	1883	15	2131	15	8096	17	14369	16
65+	2172	40	4143	39	4405	36	4979	36	14188	30	29887	34
**Comorbidity**												
No	2782	51	5297	50	6389	52	7037	51	24841	53	46346	52
Yes	2672	49	5270	50	5800	48	6803	49	22167	47	42712	48
**Comorbidity by age group†**												
<24m	73	6	148	6	187	6	202	6	841	7	1451	7
24-59m	112	18	216	19	231	18	302	21	1126	23	1987	21
5-14y	141	37	268	39	231	36	349	44	1126	43	2115	41
15-39y	145	42	361	47	479	44	625	48	2598	48	4208	47
40-64y	439	59	961	63	1183	63	1349	63	5292	65	9224	64
65+	1762	81	3316	80	3489	79	3976	80	11184	79	23727	79

† Denominator for percentages is the total number of hospitalisations in each age group for each deprivation quintile. m: months; y: years.

Influenza Vaccine Uptake

Vaccine uptake among children aged 2-4 years for the 2014/15 and 2015/16 season did not attain the DH target of 40% in most of Merseyside (Figure 4a). In 2015/16, 916/989 (93%) LSOAs did not meet the 40% target. In 2015/16. Vaccine uptake among those aged 65+ in the 2015/16 season was below the 75% DH target, with 701/989 (71%) LSOA areas below the target (Figure 4b). Among those presenting risk factors for complicated influenza, vaccine uptake in Merseyside was also below the DH target of 55%, with 853/989 (86%) LSOA areas not meeting the target in 2015/16 (Figure 4c).

Vaccine uptake was lowest in the most socioeconomically deprived quintiles (Figure 5). In the 2014/15 and 2015/16 seasons, the odds of influenza vaccine uptake in the most deprived quintile

For peer review only - http://bmjopen.bmj.com/site/about/guidelines.xhtml

compared to the least deprived were 30% lower in the 24-59 months group (OR= 0.70: 95% CI; 0.66-0.74; p<0.001), 10% lower in the 65+ year olds (OR= 0.90: 95% CI; 0.88-0.92; p<0.001). In at risk groups there was no major difference, 3% lower in most deprived (OR=1.00: 95%; CI 0.98-1.04; p=0.66).

Discussion

This study shows variations in the number of hospitalisations for IAI in Merseyside, England, across the influenza seasons from 2004/5 to 2015/16, with a significant measurable difference by socioeconomic deprivation status in the adult population, which is in concordance with findings from other settings [8,29,30]. Hospitalised IAI rates among those aged 40-64 years living in the most deprived communities were more than double those of the same age group from the most affluent areas. Influenza vaccine uptake was below the national DH target among those aged <65 years presenting comorbidities, and while it was close to the 75% target for the >65 in 20014/15, most of Merseyside was below target in 2015/16.

In contrast to our findings among adults, IAI hospitalisation rates among children <5 years of age were similar across socioeconomic strata, and vaccination uptake remained consistently below the 40% DH target for both 2014/15 and 2015/16 seasons. It could be hypothesised that in childhood, in the absence of comorbidities that could exacerbate the disease, hospitalisation following exposure to the influenza virus is likely to be driven by factors other than social determinants of health. While there is robust evidence indicating that social and economic disadvantage is strongly associated with the development of ill-health in childhood and in later life [31], our results suggest that hospitalisations for infectious respiratory diseases, such as influenza illness, are an equal threat to very young children regardless of their socioeconomic background, which is likely to be related to there being less of a differential in comorbidities across deprivation strata at a young age.

While data on vaccine uptake was not available for influenza seasons prior to 2014/15, hospitalisation rates have followed a consistent pattern throughout the study period, with an age-

For peer review only - http://bmjopen.bmj.com/site/about/guidelines.xhtml

shift observed in the 2009/10 season, consistent with the H1N1 pandemic [32]. Consistent failure to attain the DH vaccination targets across all eligible groups warrant further investigations into the perception and attitudes of the population towards vaccination in general, and the influenza vaccine specifically, and highlight the need for targeted interventions addressing low vaccine uptake among children and individuals at risk of complicated influenza to ensure national vaccination targets are met in coming years. The lower vaccine uptake rates observed among the most socioeconomically deprived populations compared the most affluent ones suggest Interventions should prioritise areas where vaccine uptake is low and hospitalisation rates are high. Vaccination with the seasonal influenza vaccine can induce long-term cross-reactive antibodies that could have a protective effect in the event of an influenza pandemic [33,34]. Highlighting this added benefit of the vaccine could potentially have a positive influence in people's perception of influenza vaccine, and contribute to improve vaccine uptake.

The strongest association between IAI hospitalisation and deprivation was seen among those aged 40-64. This could be in great part explained by the fact that unhealthy behaviours (*e.g.* smoking, alcohol abuse) and environmental risk factors (e.g. poor living environment) are associated with lower socioeconomic status. These factors will contribute cumulatively to comorbidities over time peaking in middle to old age [35], and will, in turn have a negative impact in on life expectancy, which is lower in deprived areas [36]. Interventions designed with the aim of improving the overall health and quality of life the adult population in the most socioeconomically deprived populations can potentially contribute to a reduction in IAI [37].

Limitations

Reliance on ICD-10 codes for the identification of IAI is an important limitation to our study. While we can assume that ICD-10 coding for confirmed influenza denotes laboratory confirmation, many hospitalised influenza cases will be unconfirmed by laboratory diagnostics and are likely to receive a symptom-based ICD-10 diagnosis code on discharge. Consequently, the true burden of influenza

For peer review only - http://bmjopen.bmj.com/site/about/guidelines.xhtml

hospitalisations could be higher or lower than our estimations. The use of a seasonal usage ratio to help identify ICD-10 codes associated with IAI was identified *a-priori* through professional consultation but unvalidated against a gold standard diagnostic. However, the selection of ICD-10 codes used for this study was carefully assessed and informed by previous studies [2,23,24], in an attempt to minimize misclassification. Crucially for the research aims, variations in coding over time were not observed, nor are they likely to be influenced by the socioeconomic background of the patient, since we used data from the national health system. Therefore, we are reassured that the observed association and direction of the effect have not been affected by using ICD-10 coding.

Another important limitation identified in this study is the fact that information is recorded in several databases throughout the health system without common identifiers to enable the data linkage of individuals. While hospitalisation data could be identified to LSOA level through HES, vaccine uptake could only be obtained at GP practice level through ImmForm, and was synthetically estimated at LSOA level. Moreover, none of these databases are linked to laboratory information. While the correlation with socioeconomic deprivation was demonstrated for both outcomes, and is apparent in our graphic representations, this limitation prevented the development of more complex spatio-temporal Bayesian hierarchical models. In order to evaluate interventions aimed at improving vaccine uptake and reducing the burden of hospitalisations due to IAI, surveillance data on disease incidence, hospitalisations, and vaccine uptake should be recorded in a systematic and consistent manner over time to facilitate comparison before and after the intervention was implemented. A surveillance system facilitating record matching between the various existing databases and the incorporation of laboratory data would facilitate recording disease trends and the identification of any changes in disease epidemiology, naturally or as a consequence of a public health intervention.

Finally, while our study has been able to demonstrate the association between socioeconomic deprivation and IAI hospitalisations, and with lower vaccine uptake, the results cannot elucidate the

For peer review only

For peer review only - http://bmjopen.bmj.com/site/about/guidelines.xhtml

underlying causes for this observation. Comorbidities exacerbate respiratory disease[6], and socially-deprived areas tend to have more limited access to social resources, resulting in poorer health outcomes [38]. Therefore, designing effective vaccine policy and public health interventions that translate outcomes to health equity is critical; and requires improved understanding of the healthcare system engagement that underpins lower influenza vaccine uptake [39].

Declarations

Ethical approval: The data accessed was aggregated and anonymised. Therefore, ethical approval was not required for this study

Availability of data and material: The data that support the findings of this study are available from a Public Health England but restrictions apply to the availability of these data as they are not publicly available. Aggregated data may be available from the authors/ Public Health England upon reasonable request and with permission of Public Health England.

Potential competing interests: NF and DH were in receipt of research grant support on the topic of rotavirus vaccines from GlaxoSmithKline (GSK) Biologicals. DH is in receipt of research grant support on the topic of rotavirus vaccines from Sanofi Pasteur, and Merck & Co., Inc. (Kenilworth, NJ USA) after the closure of Sanofi Pasteur-MSD in December 2016.

Funding: This study was supported by a grant from NIHR Research Capacity Funding via Liverpool NHS clinical commissioning group.

Authors' contributions: AIP, DH, PC and NF conceived of and designed the study. DH acquired and analysed the data. All authors interpreted the data; DH and AIP wrote the first draft of the report; and all authors reviewed the draft and final manuscript.

For peer review only - http://bmjopen.bmj.com/site/about/guidelines.xhtml

Acknowledgements: The authors acknowledge the contribution of Gabbie Marr at Liverpool NHS clinical commissioning group and Emer Coffey from Public Health, Liverpool City Council.

For peer review only

References

1. Pitman RJ, Melegaro A, Gelb D, Siddiqui MR, Gay NJ, Edmunds WJ. Assessing the burden of influenza and other respiratory infections in England and Wales. J Infect. 2007;54: 530–538. doi:10.1016/j.jinf.2006.09.017

2. Cromer D, van Hoek AJ, Jit M, Edmunds WJ, Fleming D, Miller E. The burden of influenza in England by age and clinical risk group: A statistical analysis to inform vaccine policy. J Infect. 2014;68: 363–371. doi:10.1016/j.jinf.2013.11.013

3. Fleming DM, Taylor RJ, Haguinet F, Schuck-Paim C, Logie J, Webb DJ, et al. Influenza-attributable burden in United Kingdom primary care. Epidemiol Infect. 2015; 1–11. doi:10.1017/S0950268815001119

4. Meier CR, Napalkov PN, Wegmüller Y, Jefferson T, Jick H. Population-based study on incidence, risk factors, clinical complications and drug utilisation associated with influenza in the United Kingdom. Eur J Clin Microbiol Infect Dis Off Publ Eur Soc Clin Microbiol. 2000;19: 834–842.

5. Pockett RD, Watkins J, McEwan P, Meier G. Burden of Illness in UK Subjects with Reported Respiratory Infections Vaccinated or Unvaccinated against Influenza: A Retrospective Observational Study. PloS One. 2015;10: e0134928. doi:10.1371/journal.pone.0134928

6. Glezen WP, Greenberg SB, Atmar RL, Piedra PA, Couch RB. Impact of respiratory virus infections on persons with chronic underlying conditions. JAMA. 2000;283: 499–505.

7. Balasegaram S, Ogilvie F, Glasswell A, Anderson C, Cleary V, Turbitt D, et al. Patterns of early transmission of pandemic influenza in London - link with deprivation. Influenza Other Respir Viruses. 2012;6: e35-41. doi:10.1111/j.1750-2659.2011.00327.x

8. Hawker JI, Olowokure B, Sufi F, Weinberg J, Gill N, Wilson RC. Social deprivation and hospital admission for respiratory infection: an ecological study. Respir Med. 2003;97: 1219–1224.

9. Rutter PD, Mytton OT, Mak M, Donaldson LJ. Socio-economic disparities in mortality due to pandemic influenza in England. Int J Public Health. 2012;57: 745–750. doi:10.1007/s00038-012-0337-1

10. Joint Committee on Vaccination and Immunisation. JCVI statement on the routine annual influenza vaccination programme - extension of the programme to children [Internet]. Department of Health; 2013 Jul pp. 1–6. Available: https://www.gov.uk/government/publications/jcvi-statement-on-the-routine-annual-influenza-vaccination-programme

11. Public Health England. Seasonal influenza vaccine uptake amongst GP Patients in England: winter season 2016 to 2017 [Internet]. London, England: Public Health England; 2017 Nov. Available: https://www.gov.uk/government/statistics/seasonal-flu-vaccine-uptake-in-gp-patients-in-england-winter-season-2016-to-2017

For peer review only

12. Public Health England. Seasonal flu vaccine uptake in children of primary school age: winter season 2016 to 2017 [Internet]. London, England: Public Health England; 2017 Nov. Available: https://www.gov.uk/government/statistics/seasonal-flu-vaccine-uptake-in-children-of-primary-school-age-winter-season-2016-to-2017

13. Public Health England. Who we are and what we do: Annual Plan 2015/16 [Internet]. London: Public Health England; 2015 Jul pp. 1–14. Available: https://www.gov.uk/government/uploads/system/uploads/attachment_data/file/452328/Annual_plan_2015-_Aug7-web.pdf

14. Hungerford D, Macpherson P, Farmer S, Ghebrehewet S, Seddon D, Vivancos R, et al. Effect of socioeconomic deprivation on uptake of measles, mumps and rubella vaccination in Liverpool, UK over 16 years: a longitudinal ecological study. Epidemiol Infect. 2015; 1–11. doi:10.1017/S0950268815002599

15. Green HK, Andrews N, Letley L, Sunderland A, White J, Pebody R. Phased introduction of a universal childhood influenza vaccination programme in England: population-level factors predicting variation in national uptake during the first year, 2013/14. Vaccine. 2015;33: 2620–2628. doi:10.1016/j.vaccine.2015.03.049

16. Spencer AM, Roberts SA, Brabin L, Patnick J, Verma A. Sociodemographic factors predicting mother's cervical screening and daughter's HPV vaccination uptake. J Epidemiol Community Health. 2014;68: 571–577. doi:10.1136/jech-2013-202629

17. Baguelin M, Flasche S, Camacho A, Demiris N, Miller E, Edmunds WJ. Assessing Optimal Target Populations for Influenza Vaccination Programmes: An Evidence Synthesis and Modelling Study. PLoS Med. 2013;10: e1001527. doi:10.1371/journal.pmed.1001527

18. Baguelin M, Hoek AJV, Jit M, Flasche S, White PJ, Edmunds WJ. Vaccination against pandemic influenza A/H1N1v in England: a real-time economic evaluation. Vaccine. 2010;28: 2370–2384. doi:10.1016/j.vaccine.2010.01.002

19. Hiscock R, Bauld L, Amos A, Platt S. Smoking and socioeconomic status in England: the rise of the never smoker and the disadvantaged smoker. J Public Health Oxf Engl. 2012;34: 390–396. doi:10.1093/pubmed/fds012

20. Newton JN, Briggs ADM, Murray CJL, Dicker D, Foreman KJ, Wang H, et al. Changes in health in England, with analysis by English regions and areas of deprivation, 1990-2013: a systematic analysis for the Global Burden of Disease Study 2013. Lancet Lond Engl. 2015;386: 2257–2274. doi:10.1016/S0140-6736(15)00195-6

21. Office for National Statistics. Lower Super Output Area Mid-Year Population Estimates [Internet]. 26 Oct 2016 [cited 5 Jul 2017]. Available: https://www.ons.gov.uk/peoplepopulationandcommunity/populationandmigration/populationestimates/datasets/lowersuperoutputareamidyearpopulationestimates

22. Department for Communities and Local Government. English indices of deprivation 2015 [Internet]. London, England: Department for Communities and Local Government; 2015 Sep. Available: https://www.gov.uk/government/statistics/english-indices-of-deprivation-2015

For peer review only - http://bmjopen.bmj.com/site/about/guidelines.xhtml

23. Moore HC, Lehmann D, Klerk N de, Smith DW, Richmond PC, Keil AD, et al. How Accurate Are International Classification of Diseases-10 Diagnosis Codes in Detecting Influenza and Pertussis Hospitalizations in Children? J Pediatr Infect Dis Soc. 2014;3: 255–260. doi:10.1093/jpids/pit036

24. Moore K, Black J, Rowe S, Franklin L. Syndromic surveillance for influenza in two hospital emergency departments. Relationships between ICD-10 codes and notified cases, before and during a pandemic. BMC Public Health. 2011;11: 338. doi:10.1186/1471-2458-11-338

25. Buda S, Tolksdorf K, Schuler E, Kuhlen R, Haas W. Establishing an ICD-10 code based SARI-surveillance in Germany - description of the system and first results from five recent influenza seasons. BMC Public Health. 2017;17: 612. doi:10.1186/s12889-017-4515-1

26. Public Health England. Flu plan: winter 2016 to 2017 [Internet]. London, England: Public Health England; 2017 May. Available: https://www.gov.uk/government/publications/flu-plan-winter-2016-to-2017

27. Papke LE, Wooldridge JM. Econometric methods for fractional response variables with an application to 401(k) plan participation rates. J Appl Econom. 1996;11: 619–632. doi:10.1002/(SICI)1099-1255(199611)11:6<619::AID-JAE418>3.0.CO;2-1

28. R Core Team. R: A Language and Environment for Statistical Computing [Internet]. Vienna, Austria: R Foundation for Statistical Computing; 2016. Available: http://www.R-project.org

29. Charland KM, Brownstein JS, Verma A, Brien S, Buckeridge DL. Socio-economic disparities in the burden of seasonal influenza: the effect of social and material deprivation on rates of influenza infection. PloS One PLoS ONE. 2011;6, 6: e17207–e17207. doi:10.1371/journal.pone.0017207, 10.1371/journal.pone.0017207

30. Hyder A, Leung B. Social deprivation and burden of influenza: Testing hypotheses and gaining insights from a simulation model for the spread of influenza. Epidemics. 2015;11: 71–79. doi:10.1016/j.epidem.2015.03.004

31. Wickham S, Anwar E, Barr B, Law C, Taylor-Robinson D. Poverty and child health in the UK: using evidence for action. Arch Dis Child. 2016;101: 759. doi:10.1136/archdischild-2014-306746

32. Falagas ME, Koletsi PK, Baskouta E, Rafailidis PI, Dimopoulos G, Karageorgopoulos DE. Pandemic A(H1N1) 2009 influenza: review of the Southern Hemisphere experience. Epidemiol Infect. 2011;139: 27–40. doi:10.1017/S0950268810002037

33. McCullers JA, Van De Velde L-A, Allison KJ, Branum KC, Webby RJ, Flynn PM. Recipients of vaccine against the 1976 “swine flu” have enhanced neutralization responses to the 2009 novel H1N1 influenza virus. Clin Infect Dis Off Publ Infect Dis Soc Am. 2010;50: 1487–1492. doi:10.1086/652441

For peer review only

For peer review only - http://bmjopen.bmj.com/site/about/guidelines.xhtml

34. Wei C-J, Boyington JC, Dai K, Houser KV, Pearce MB, Kong W-P, et al. Cross-neutralization of 1918 and 2009 influenza viruses: role of glycans in viral evolution and vaccine design. Sci Transl Med. 2010;2: 24ra21. doi:10.1126/scitranslmed.3000799

35. Bonnie RJ, Stratton K, Kwan LY, Products C on the PHI of R the MA for PT, Practice B on PH and PH, Medicine I of. The Effects of Tobacco Use on Health [Internet]. National Academies Press (US); 2015. Available: https://www.ncbi.nlm.nih.gov/books/NBK310413/

36. Health state life expectancies by Index of Multiple Deprivation (IMD) - Office for National Statistics [Internet]. [cited 22 Jan 2018]. Available: https://www.ons.gov.uk/peoplepopulationandcommunity/healthandsocialcare/healthinequalities/bulletins/healthstatelifeexpectanciesbyindexofmultipledeprivationimd/england2013to2015

37. Lawson KD, Mercer SW, Wyke S, Grieve E, Guthrie B, Watt GC, et al. Double trouble: the impact of multimorbidity and deprivation on preference-weighted health related quality of life a cross sectional analysis of the Scottish Health Survey. Int J Equity Health. 2013;12: 67. doi:10.1186/1475-9276-12-67

38. Zhang X, Cook PA, Lisboa PJ, Jarman IH, Bellis MA. The effects of deprivation and relative deprivation on self-reported morbidity in England: an area-level ecological study. Int J Health Geogr. 2013;12: 5. doi:10.1186/1476-072X-12-5

39. Nagata JM, Hernández-Ramos I, Kurup AS, Albrecht D, Vivas-Torrealba C, Franco-Paredes C. Social determinants of health and seasonal influenza vaccination in adults ≥65 years: a systematic review of qualitative and quantitative data. BMC Public Health. 2013;13: 388. doi:10.1186/1471-2458-13-388

For peer review only

For peer review only - http://bmjopen.bmj.com/site/about/guidelines.xhtml

Figure 1. Number of hospitalisations for IAI by Merseyside residents, 5 week moving average 2004/05-2015/16, by age-groups. m: months; y: years

Figure 2. Incidence rate ratios of IAI hospitalisation rate for Index of Multiple Deprivation quintiles 1 (most deprived)-4 compared to a reference group of the least deprived quintile (5) by age group

Rate ratios for IAI hospitalised incidence rates were estimated with negative binomial regression stratified by age group. Models were additionally adjusted for sex and fiscal year. m: months; y: years. * $p < 0.05$, ** $p < 0.01$, ***$p < 0.001$

Figure 3. Age and sex standardised incidence ratios for IAI hospitalisations for Merseyside residents by MSOA and pooled years, 2004/05 - 2015/16

Figure 4. Influenza vaccine uptake by LSOA 2014/15 and 2015/16 seasons in: a) 24-59 month olds (40% NHS England target); b) 65+ year olds (75% NHS England target); c) at risk groups aged 6 months to 64 years (55% NHS England target). Each block represents 1 of 989 LSOAs in Merseyside.

Figure 5. Odds ratios of vaccine uptake by Index of Multiple Deprivation quintiles 1 (most deprived)-4 compared to a reference group of the least deprived quintile (5) by age group, 2014/15-2015/16

Adjusted odds ratios for vaccine uptake were estimated with general linear models of the binomial family with a logit function, robust standard errors were used to calculate 95% confidence intervals. Models were additionally adjusted for fiscal year and weighted using the relevant age related population of each LSOA. m, months.

* $p < 0.05$, ** $p < 0.01$, ***$p < 0.001$

For peer review only - http://bmjopen.bmj.com/site/about/guidelines.xhtml

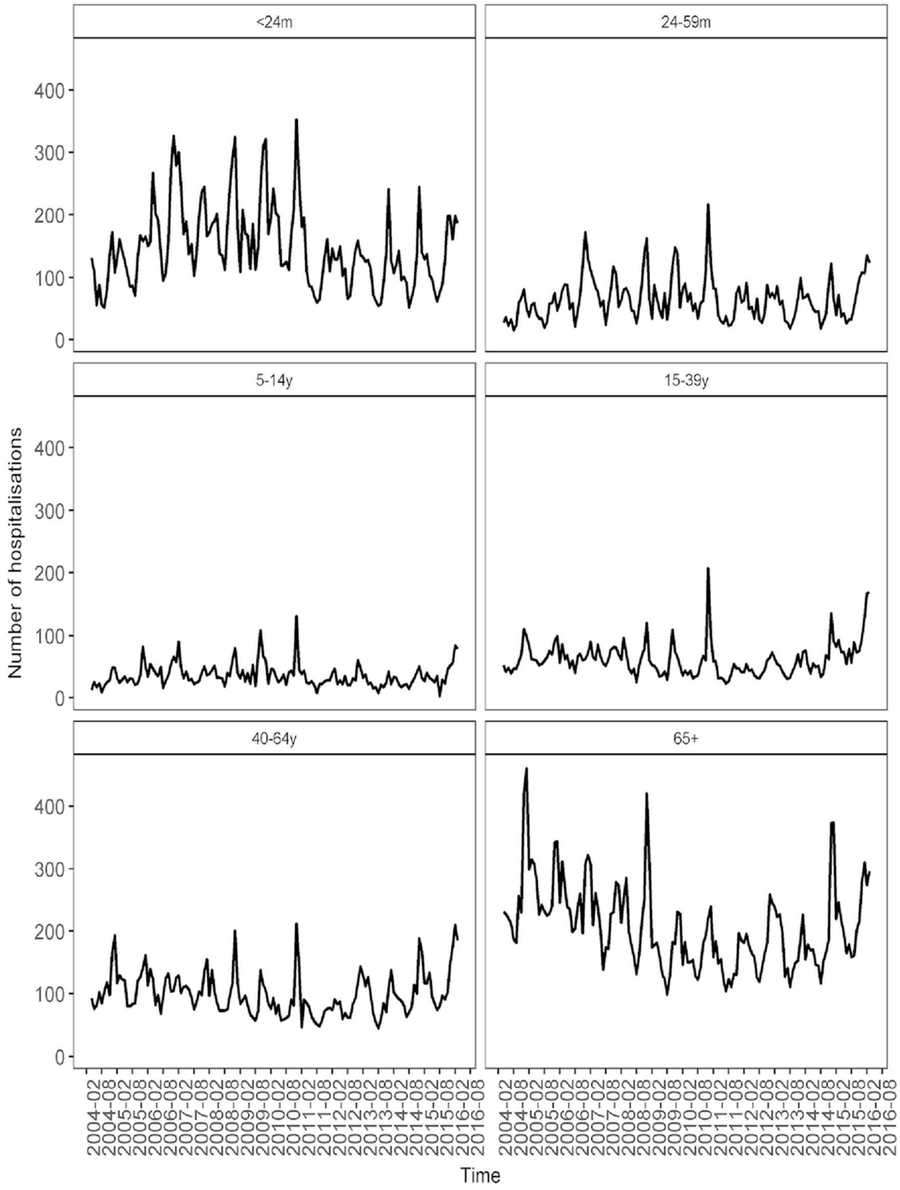

Figure 1. Number of hospitalisations for IAI by Merseyside residents, 5 week moving average 2004/05-2015/16, by age-groups

99x133mm (300 x 300 DPI)

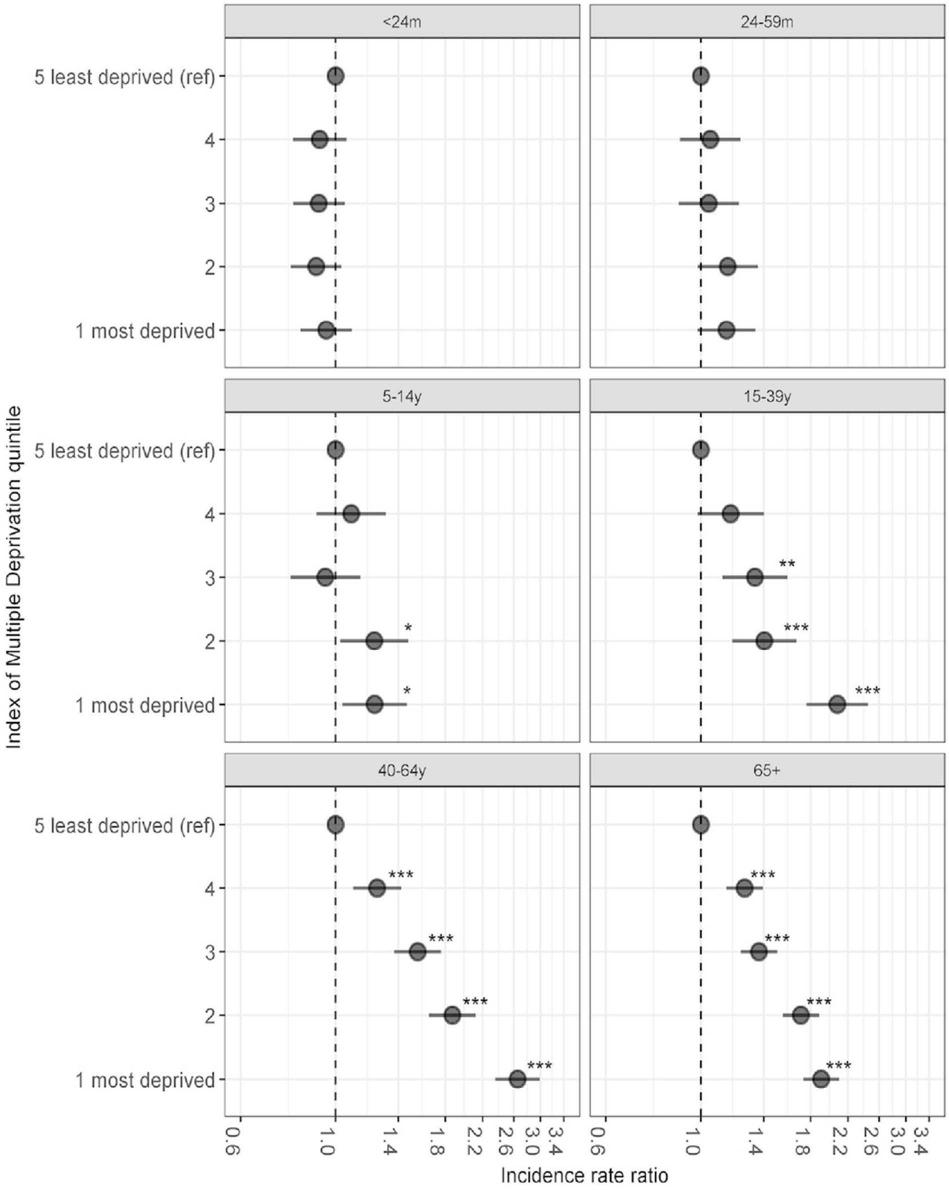

Figure 2. Incidence rate ratios of IAI hospitalisation rate for Index of Multiple Deprivation quintiles 1 (most deprived)-4 compared to a reference group of the least deprived quintile (5) by age group

90x112mm (300 x 300 DPI)

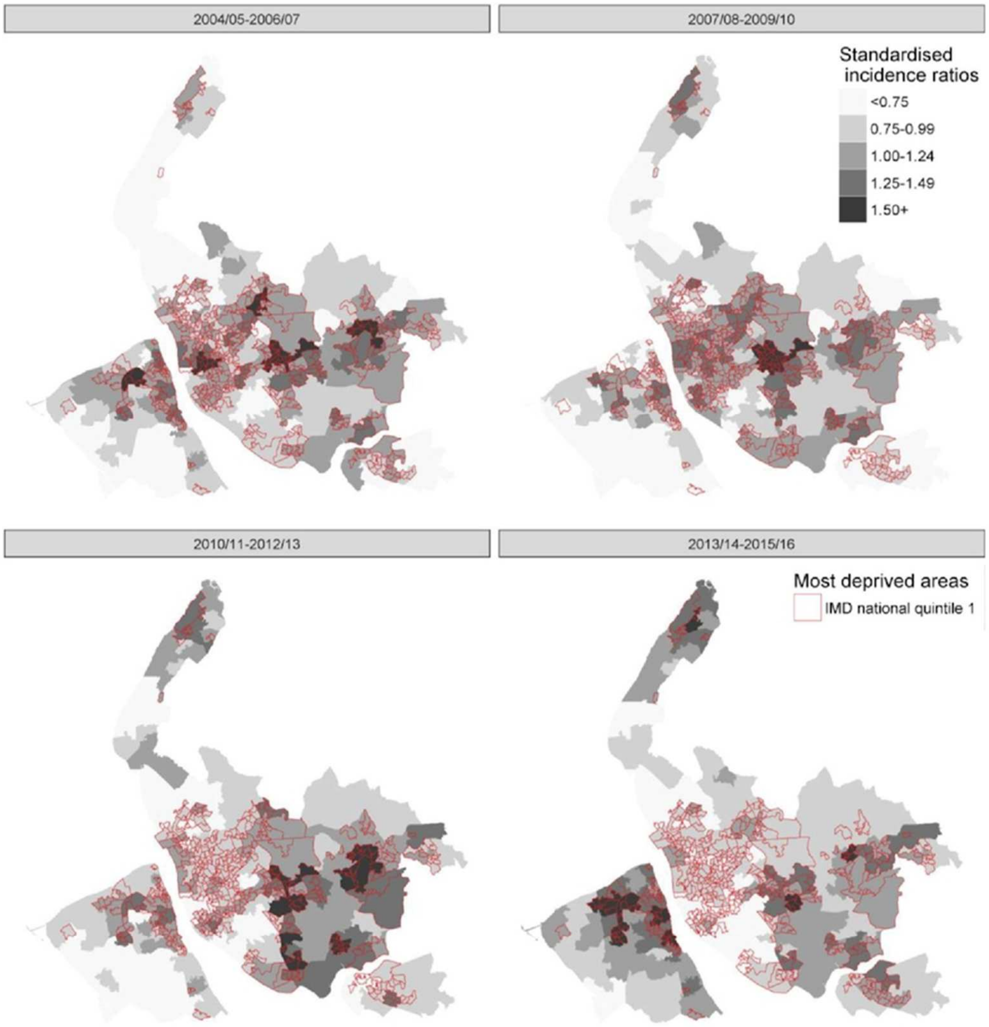

Figure 3. Age and sex standardised incidence ratios for IAI hospitalisations for Merseyside residents by MSOA and pooled years, 2004/05 - 2015/16

75x78mm (300 x 300 DPI)

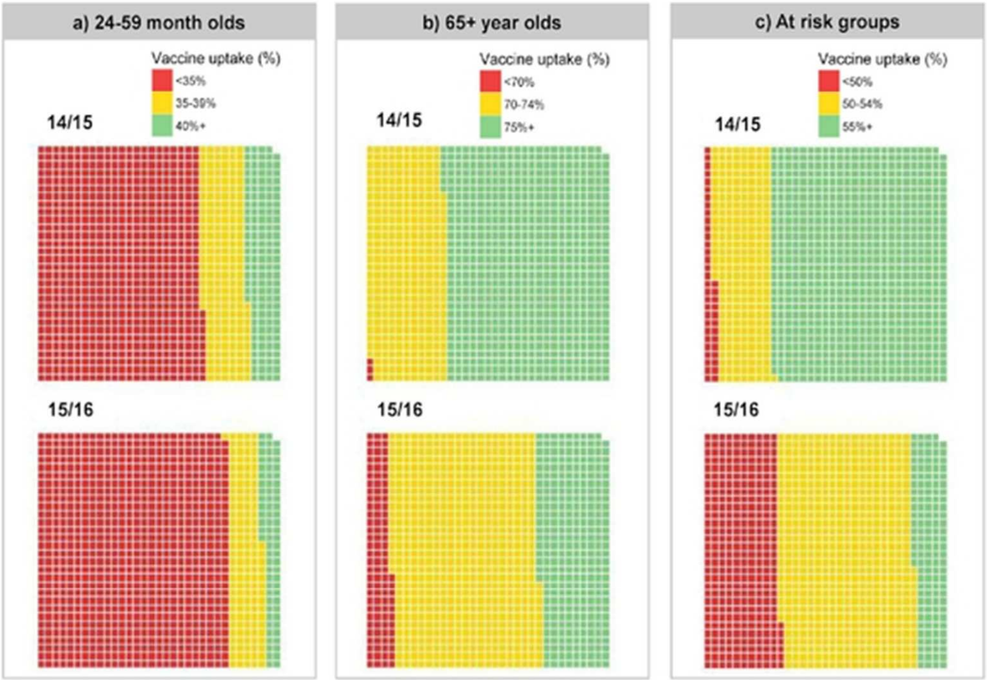

Figure 4. Influenza vaccine uptake by LSOA 2014/15 and 2015/16 seasons in: a) 24-59 month olds (40% NHS England target); b) 65+ year olds (75% NHS England target); c) at risk groups aged 6 months to 64 years (55% NHS England target)

50x34mm (300 x 300 DPI)

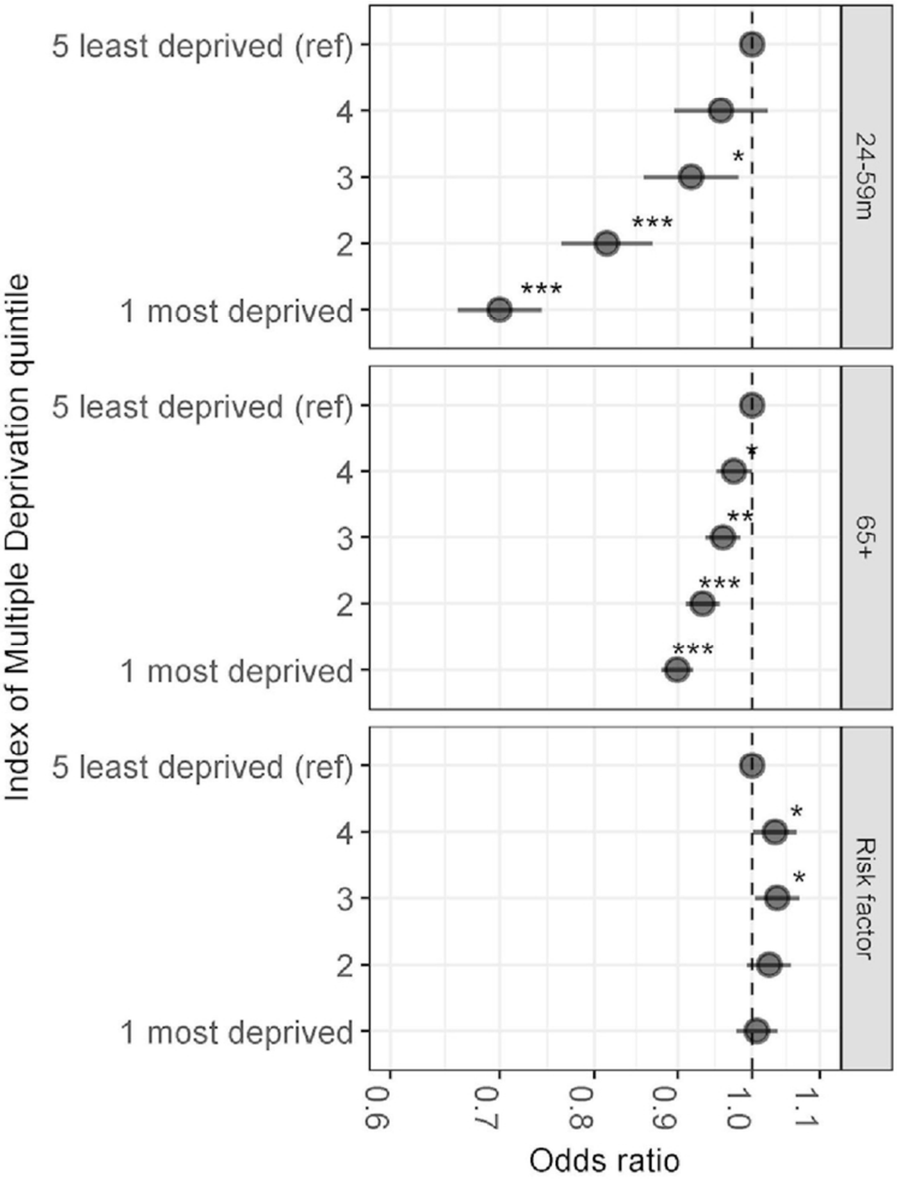

Figure 5. Odds ratios of vaccine uptake by Index of Multiple Deprivation quintiles 1 (most deprived)-4 compared to a reference group of the least deprived quintile (5) by age group, 2014/15-2015/16

65x84mm (300 x 300 DPI)

S1-Table 1. ICD-10 codes used to identify comorbidities adapted from Cromer et al. 2014

Condition	ICD-10 codes used
**Chronic respiratory disease**	J4, J6, J7, J8, Q30, J31, Q32, Q33Q34, Q35, Q36, Q37
**Chronic heart disease**	I05, I06, I07, I08, I09, I11, I12, I13, I20, I21, I22, I25, I27, I28, I3, I40, I41, I42, I43, I44, I45, I47, I48, I49, I5, I6, Q2
**Chronic kidney disease**	N0, N11, N12, N14, N15, N16, N18, N19, N25, Q60, Q61
**Chronic liver disease**	K7, P78.8, Q44, B18
**Chronic neurological disease**	G1, G2, G3, G4, G5, G6, G7, G8, G9
**Diabetes**	E10, E11, E12, E13, E14, E24, G59.0, G63.2, G73.0, G99.0, N08.3, O24, P70.0, P70.1, P70.2
**Immunosuppression**	All C-codes, D37, D38, D39, D4, B20, B21, B22, B23, B24, Z94, Z85, Z94.8, D56.1, D57.8, D57.0, D57.D61, D70, D71, D72, D73, D76, D80, D81, D82, D83, D84, K90.0
**Other including: asplenia or dysfunction of the spleen, cochlear implants and cerebrospinal fluid leaks**	D73, D56.1, D57.8, D57.0, D57.1, K90.0, Z96.1, G96.0

For peer review only

S1-Table 2. Demographics of Merseyside residents admitted to hospital with IAI (ICD-10: J09, J10, J128, J129, J11, J22, J069) by financial year, 2004/05 to 2015/16 (N=89,058)

	04/05 (N=7457)		05/06 (N=8018)		06/07 (N=9248)		07/08 (N=8175)		08/09 (N=8269)		09/10 (N=7559)		10/11 (N=7640)		11/12 (N=5231)		12/13 (N=6399)		13/14 (N=5883)		14/15 (N=6985)		15/16 (N=8194)	
Sex	n	%	n	%	n	%	n	%	n	%	n	%	n	%	n	%	n	%	n	%	n	%	n	%
Female	3900	52	4173	52	4572	49	4069	50	4171	50	3759	50	3777	49	2633	50	3267	51	3048	52	3730	53	4190	51
Male	3556	48	3845	48	4675	51	4106	50	4098	50	3800	50	3863	51	2598	50	3132	49	2835	48	3255	47	4004	49
Unknown	1	0	0	0	1	0	0	0	0	0	0	0	0	0	0	0	0	0	0	0	0	0	0	0
**Age group**																								
<24m	1281	17	1544	19	2583	28	2089	26	2310	28	2376	31	2235	29	1250	24	1441	23	1315	22	1425	20	1572	19
24-59m	510	7	597	7	1057	11	857	10	908	11	954	13	1013	13	589	11	692	11	643	11	680	10	873	11
5-14y	309	4	449	6	565	6	412	5	492	6	575	8	523	7	313	6	383	6	277	5	340	5	490	6
15-39y	752	10	825	10	761	8	844	10	708	9	628	8	785	10	463	9	581	9	605	10	797	11	1131	14
40-64y	1363	18	1376	17	1290	14	1312	16	1186	14	1049	14	1057	14	829	16	1104	17	1053	18	1277	18	1473	18
65+	3242	43	3227	40	2992	32	2661	33	2665	32	1977	26	2027	27	1787	34	2198	34	1990	34	2466	35	2655	32
**National IMD quintile**																								
5 (least deprived)	410	5	468	6	511	6	449	5	497	6	400	5	452	6	348	7	427	7	384	7	514	7	594	7
4	855	11	946	12	1005	11	959	12	914	11	831	11	862	11	654	13	837	13	764	13	880	13	1060	13
3	983	13	1107	14	1255	14	1038	13	1107	13	986	13	1007	13	762	15	996	16	856	15	998	14	1094	13
2	1151	15	1250	16	1445	16	1193	15	1292	16	1096	14	1096	14	818	16	1013	16	940	16	1149	16	1397	17
1 (most deprived)	4058	54	4247	53	5032	54	4536	55	4459	54	4246	56	4223	55	2649	51	3126	49	2939	50	3444	49	4049	49
**Comorbidity**																								
**No**	3617	49	4109	51	5442	59	4709	58	4528	55	4357	58	4360	57	2553	49	3046	48	2757	47	3073	44	3795	46
**Yes**	3840	51	3909	49	3806	41	3466	42	3741	45	3202	42	3280	43	2678	51	3353	52	3126	53	3912	56	4399	54

IMD: Index of Multiple Deprivation; m: months; y: years

S1-Table 3. Interaction between socioeconomic deprivation (IMD quintile) and age group on the rate of ILI hospitalisation in Merseyside, UK

	Mean yearly rate per 1,000 population	IRR	Full model with interaction: IRR	Standard error	P value
**Main effects**					
National IMD quintile					
5 (least deprived)	3.46	Reference	Reference		
4	3.98	1.13 (1.05-1.21)	1.00 (0.86-1.16)	0.077	0.984
3	4.26	1.18 (1.10-1.27)	0.99 (0.85-1.15)	0.076	0.864
2	4.82	1.37 (1.27-1.47)	1.04 (0.90-1.21)	0.076	0.581
1 (most deprived)	5.84	1.61 (1.50-1.72)	1.1 (0.95-1.27)	0.074	0.19
Age group					
<24m (reference)	51.3	Reference	Reference		
24-59m	15.4	0.30 (0.28-0.32)	0.29 (0.24-0.34)	0.084	<0.001
5-14y	2.49	0.05 (0.04-0.05)	0.05 (0.04-0.05)	0.090	<0.001
15-39y	1.50	0.03 (0.02-0.03)	0.02 (0.02-0.02)	0.091	<0.001
40-64y	2.43	0.04 (0.04-0.04)	0.03 (0.02-0.03)	0.082	<0.001
65+	9.66	0.18 (0.16-0.19	0.13 (0.11-0.15)	0.077	<0.001
Year	4.94	0.99 (0.98-1.00)	0.99 (0.98-1.00)	0.003	<0.001
Sex					
Male	5.01	Reference	Reference		
Female	4.88	0.98 (0.94-1.02)	0.98 (0.94-1.02)	0.020	0.263
**Interaction effects (IMD quintile interacted with age group)**					
IMD quintile 5 * <24m			reference		
IMD quintile 4 * 24-59m	-	-	1.05 (0.84-1.31)	0.114	0.663
IMD quintile 3 * 24-59m	-	-	1.06 (0.85-1.32)	0.113	0.628
IMD quintile 2* 24-59m	-	-	1.10 (0.89-1.38)	0.113	0.38
IMD quintile 1 * 24-59m	-	-	1.04 (0.84-1.29)	0.109	0.736
IMD quintile 4 * 5-14y	-	-	1.08 (0.85-1.37)	0.121	0.524
IMD quintile 3 * 5-14y	-	-	0.95 (0.75-1.21)	0.121	0.7
IMD quintile 2 * 5-14y	-	-	1.17 (0.93-1.48)	0.119	0.187
IMD quintile 1 * 5-14y	-	-	1.11 (0.88-1.39)	0.114	0.375
IMD quintile 4 * 15-39y	-	-	1.17 (0.92-1.48)	0.121	0.205
IMD quintile 3 * 15-39y	-	-	1.33 (1.06-1.68)	0.119	0.016
IMD quintile 2 * 15-39y	-	-	1.34 (1.06-1.69)	0.118	0.014
IMD quintile 1 * 15-39y	-	-	1.88 (1.50-2.35)	0.115	<0.001
IMD quintile 4 * 40-64y	-	-	1.25 (1.00-1.55)	0.112	0.05
IMD quintile 3 * 40-64y	-	-	1.57 (1.26-1.95)	0.111	<0.001
IMD quintile 2 * 40-64y	-	-	1.79 (1.44-2.22)	0.110	<0.001
IMD quintile 1 * 40-64y	-	-	2.39 (1.94-2.95)	0.108	<0.001
IMD quintile 4 * 65+	-	-	1.26 (1.03-1.56)	0.106	0.026
IMD quintile 3 * 65+	-	-	1.39 (1.13-1.71)	0.105	0.002
IMD quintile 2 * 65+	-	-	1.65 (1.34-2.02)	0.105	<0.001
IMD quintile 1 * 65+	-	-	1.75 (1.43-2.14)	0.103	<0.001

IMD: Index of Multiple deprivation; IRR: Incidence rate ratio; m: months; y: years

For peer review only

BMJ Open

Influenza associated hospitalisation, vaccine uptake and socioeconomic deprivation in an English city region: an ecological study

Journal:	*BMJ Open*
Manuscript ID	bmjopen-2018-023275.R1
Article Type:	Research
Date Submitted by the Author:	08-Aug-2018
Complete List of Authors:	Hungerford, Daniel; University of Liverpool, Centre for Global Vaccine Research, Institute of Infection and Global Health; Public Health England, Field Epidemiology Service, Health Protection<br>Ibarz-Pavon, Ana; University of Liverpool, Centre for Global Vaccine Research, Institute of Infection and Global Health<br>Cleary, Paul; Public Health England, Field Epidemiology Service<br>French, Neil; University of Liverpool, Centre for Global Vaccine Research, Institute of Infection and Global Health; Malawi-Liverpool-Wellcome Trust Clinical Research Programme, College of Medicine, University of Malawi
**Primary Subject Heading**:	Infectious diseases
Secondary Subject Heading:	Public health, Epidemiology, General practice / Family practice
Keywords:	Epidemiology < INFECTIOUS DISEASES, Public health < INFECTIOUS DISEASES, Inequalities, Influenza, Vaccination, ecological

SCHOLARONE™
Manuscripts

Influenza associated hospitalisation, vaccine uptake and socioeconomic deprivation in an English city region: an ecological study

Daniel Hungerford[1,2]*, Ana Ibarz-Pavon[1], Paul Cleary[2], Neil French[1]

1. The Centre for Global Vaccine Research, Institute of Infection and Global Health, University of Liverpool, Liverpool, UK

2. Field Epidemiology Service, National Infection Service, Public Health England, Liverpool, UK

Word count=3904

*Correspondence: d.hungerford@liverpool.ac.uk

Short title: Hospitalised influenza associated illness, vaccine uptake and deprivation in the UK

For peer review only

Abstract

Objectives: Every year, influenza poses a significant burden on the National Health Service in England. Influenza vaccination is an effective measure to prevent severe disease, hence, maximising vaccine coverage in the most vulnerable is a priority. We aimed to identify the extent to which socioeconomic status is associated with influenza-associated illness (IAI) and influenza vaccine coverage.

Design: Retrospective observational study using hospital episode statistics.

Setting: Merseyside, North-West of England, including the city of Liverpool.

Participants: Residents of Merseyside hospitalised with IAI between April 2004 and March 2016, and Merseyside general practice registered patients eligible for influenza vaccination in 2014/15 and 2015/16 influenza seasons.

Exposures: Socioeconomic deprivation based on lower super output area English Indices of Deprivation scores.

Primary and secondary outcome measures: Incidence and risk of IAI hospitalisation, and vaccine uptake.

Results: There were 89,058 hospitalisations related to IAI among Merseyside residents (mean yearly rate = 4.9 per 1,000 population). Hospitalisations for IAI were more frequent in the most socioeconomically deprived areas compared to the least deprived in adults aged 15-39 years (incidence rate ratio [IRR]=2.08: 95% CI; 1.76-2.45; p<0.001), 60-64 years (IRR=2.65: 95% CI; 2.35-2.99; p<0.001) and 65+ years (IRR=1.90: 95% CI; 1.73-2.10; p<0.001); whereas rates in children were more homogeneous across deprivation strata. Vaccine uptake was lower than the nationally-set targets in most neighbourhoods. The odds of vaccine uptake were 30% lower (odds ratio [OR]= 0.70: 95% CI; 0.66-0.74; p<0.001) and 10% lower (OR= 0.90: 95% CI; 0.88-0.92; p<0.001) in the most deprived IMD quintile compared to the least deprived among children aged 24-59 months and 65+

For peer review only - http://bmjopen.bmj.com/site/about/guidelines.xhtml

year olds, respectively.

Conclusions: Higher rates of IAI hospitalisations and lower vaccine uptake in the most socioeconomically deprived populations suggest that health promotion policies and interventions that target these populations should be a priority.

Strengths and limitations of study methodology

- The study uses routine public health data sources and a simple repeatable methodology which can be applied to continue to monitor changes in incidence and vaccine uptake in local areas, and potentially evaluate targeted population-level interventions.
- We were unable to evaluate population-level impact of influenza vaccines because of non-specific endpoints and seasonal disease variation over the study period.
- While an association with socioeconomic deprivation was demonstrated for incidence of hospitalisation and uptake independently, difficulties linking data recorded through several incompatible databases prevented integration and development of more complex models.
- Further research is needed to understand the attitudes and beliefs toward influenza disease and influenza vaccination as this will influence vaccine acceptability and uptake among the population

For peer review only - http://bmjopen.bmj.com/site/about/guidelines.xhtml

Background

Influenza is a highly infectious disease associated with high morbidity and mortality rates among vulnerable populations worldwide. Although influenza imposes a considerable burden on the National Health Service (NHS) in the United Kingdom (UK), estimating the true burden of disease is difficult due to the non-specific symptomatic presentation of disease and lack of laboratory confirmation. Using routinely collected healthcare system and mortality data, influenza was estimated to account for almost 900,000 GP consultations, 25,000 hospitalisations and 20,000 deaths in England and Wales every year [1]. However, several studies attempting to estimate the true burden of disease using different approaches and statistical modelling suggest that burden of illness attributable to influenza in the UK is likely to be underestimated by current surveillance systems [1–5]. Children <5 years of age and those aged 65+ years and individuals with underlying comorbidities are known to be at higher risk of complicated influenza [2,6].Socioeconomic deprivation has also been associated with higher influenza-related mortality and hospitalisations [7–9].

Vaccination is the most effective control measure against complications of seasonal influenza. In the UK, the vaccine is offered free of charge through the NHS to those aged 65 and over, and to individuals at risk of complicated influenza under 65 years of age. In 2013, the Department of Health (DH) expanded free vaccination to children with an age-phased rollout of an intranasal Live Attenuated Influenza Vaccine (LAIV) programme, that at the time of writing, includes all children aged 2-8 years, and will eventually target all those up to 17 years of age [10]. Children aged 2-4 years receive the vaccine at their general practice (GP) while those aged 5+ years are vaccinated at school. During the 2016/17 influenza season, vaccine uptake in England was 70.5% among those aged 65+ years, 36.9% among children aged 2-4 years, 48.6% in risk groups aged 6 months to 64 years and 55.4% among children aged 5-8 years in school years 1 to 3 [11,12]. However, there is variation in uptake between GP practices and few practices achieve the 40% LAIV uptake target in 2-4 year olds [13]. There is an inverse association between deprivation and uptake of routine childhood vaccines

For peer review only - http://bmjopen.bmj.com/site/about/guidelines.xhtml

(*e.g.* vaccines for measles, mumps and rubella, human papillomavirus, influenza) [14–16]. Therefore, optimizing vaccine delivery and identifying key target areas is necessary to ensure the protection of those most vulnerable, and to maximize cost-effectiveness. Simulation studies supporting the decision to include LAIV in the vaccination schedule indicated that increasing vaccine uptake in children from 30% to 60% can markedly increase both direct and indirect protective effects of the vaccine [1,17,18]. However, these studies assume uniform vaccine coverage and fail to account for the substantial variation in disease risk and vaccine uptake observed across local areas with different socioeconomic status [15]. Given that comorbidities that increase the risk of adverse outcomes from influenza are substantially more prevalent among the most deprived populations [19,20], it would be expected that ensuring vaccine delivery to the most vulnerable groups would increase cost-effectiveness. Understanding how vaccine uptake varies across local areas with differing socioeconomic status, and correlating this to disease burden and use of healthcare resources, will provide evidence to inform immunisation policy and assist in developing targeted vaccine interventions to mitigate this effect. The aim of this study was to describe the burden of severe influenza associated illness (IAI) in secondary care across local communities in a defined population in relation to small area level socioeconomic deprivation and vaccine uptake levels. This information will help to identify priority populations for public health interventions to increase vaccine uptake and to reduce the health and economic impact of influenza.

Methods

Population and Setting

The study population was the metropolitan area of Merseyside, North-West England, defined in this study as the local authority areas of Halton, Knowsley, Liverpool, Sefton, St. Helens, and Wirral. Merseyside has an estimated resident population of 1.5 million [21], and is a diverse area that contains some of the most socioeconomically deprived neighbourhoods in England as well as some of the most affluent [22].

For peer review only

Data sources

Influenza vaccine uptake

Influenza vaccine uptake data for seasons 2014/15 and 2015/16 were obtained from ImmForm, the system used by the DH, the NHS, and Public Health England (PHE) to record GP vaccination uptake [21]. In addition to vaccine delivery through GP's, since 2015/2016 pharmacies in England have also been able to offer influenza vaccination [23]. To capture these data, pharmacies are required to report any vaccine administration to the patients registered GP, and the GP will input this into the ImmForm system. We extracted aggregated information on vaccine uptake for all groups eligible for vaccination from 288 GP practices in Merseyside. Data included the number of eligible patients and the number of people receiving the vaccine for each eligible group.

Hospitalisations for influenza-associated illness

Hospitalisation data were extracted from the national Hospital Episode Statistics (HES) database via PHE, a records-based system maintained by NHS digital, which contains information on all hospital admissions, outpatient appointments, and A&E attendances covering all NHS trusts in England.

We extracted anonymised aggregated records of all patients residing in Merseyside admitted as an emergency to a hospital in England with a primary or subsequent diagnosis of acute respiratory illness identified between April 2004 and March 2016. We used the International Statistical Classification of Diseases and Related Health Problems (ICD-10) codes (ICD-10 codes J0*, J1*, J2*, J3*) to identify acute respiratory infection [2]. We further used the ICD10-coding to identify confirmed influenza, IAI, and comorbidities. Confirmed influenza was identified by codes J09 and J10. IAI codes were selected using the following procedure and criteria:

- Literature search of peer-reviewed publications [2,24,25]
- Identification of ICD-10 codes used by the NHS trusts as a primary diagnostic of respiratory diseases during the peak influenza season (December to March). We calculated means of daily usage during the peak influenza compared to the rest of year

For peer review only - http://bmjopen.bmj.com/site/about/guidelines.xhtml

and excluded codes which had a daily usage ratio of less than three during the peak influenza season compared to the rest of the year [26].

- Excluding specific ICD-10 codes which were identifying non-influenza related illness, such as, for example, J211-RSV pneumonia, J123-Human Metapneumovirus pneumonia and J301-Allergic rhinitis due to pollen.
- Excluding ICD-10 codes (J218 and J219) commonly used for Respiratory Syncytial Virus (RSV). We identified these codes through a prior study at a major acute care hospital in Merseyside by cross-checking patients with laboratory confirmed RSV with their ICD-10 primary diagnosis code on discharge (Heinsbroek E, The impact of rotavirus vaccination on hospital pressures in a large paediatric hospital in the United Kingdom, ClinicalTrials.gov NCT03271593).

Final selected ICD-10 codes for IAI were as follows: J06.9 (unspecified acute upper respiratory tract infection), J09 (influenza due to certain identified influenza virus), J10 (Influenza due to other identified influenza virus), J11 (Influenza, virus not identified), J12.8 (Other viral pneumonia), J12.9 (Viral pneumonia, unspecified), J22 (unspecified acute lower respiratory tract infection). ICD-10 codes used to identify comorbidities are listed in supplementary S1-Table 1 and were identified using the PHE Flu Plan 2016/17 and Cromer *et al.* 2014 [2,27].

Area of residence, population denominators and socioeconomic deprivation

The HES data set includes a code for neighbourhood areas of residence, known as Lower Super Output Areas (LSOAs). There are 32,482 LSOAs in England, each containing approximately 1,500 persons [21]. These statistical boundaries were constructed after the 2011 census, and have an associated measure of socioeconomic deprivation: the English Indices of Deprivation 2015, of which the Index of Multiple Deprivation (IMD) is the most commonly used. The IMD is a composite measure of social deprivation based on seven weighted domains: income; employment; health and

For peer review only - http://bmjopen.bmj.com/site/about/guidelines.xhtml

disability; education skills and training; barriers to housing and other services; crime; and living environment [22]. Population denominators at LSOA and middle super output area (MSOA) were accessed for 2004 to 2015 by single year of age and sex and aggregated into the appropriate age categories referred to below. This information is published by the Office for National Statistics (ONS) and held by PHE [21].

Statistical analysis

Hospitalisation data from HES were recoded, reshaped, and aggregated by age, sex, and time before merging to other data sources. Age categories were constructed based on age at hospitalisation: <24m; 24-59m; 5-14y; 15-39y; 40-64y; 65+.

To analyse the spatial and temporal changes in IAI hospitalisations, we constructed age and sex adjusted standardised incidence ratios (SIRs) for Merseyside at the MSOA level (approximately 5,000-12,000 resident population) to avoid small numbers. SIRs that represent the relative risk of IAI hospitalisation in an MSOA were compared to the Merseyside average. To calculate SIRs, we first calculated the expected number of hospitalisations in each $MSOA_{ijk}$ by calculating overall Merseyside rates for each combination of age $_j$ and sex $_k$ and multiplying these by corresponding MSOA population denominators, as exampled in the following equation:

$$Expected\ number_{ijk} = \frac{MSOA\ population_{jk}}{Total\ population_{jk}} \times Total\ number\ of\ hospitalisations_{jk}$$

Expected numbers are summed for each MSOA and SIRs are calculated by the ratio of the observed to expected numbers in each MSOA, as shown below:

$$SIR_i = \frac{Observed\ number\ of\ hospitalisations_i}{Expected\ number\ of\ hospitalisations_i}$$

We then produced choropleth maps of MSOA SIR for four three-year pooled time periods for the area of Merseyside.

For peer review only - http://bmjopen.bmj.com/site/about/guidelines.xhtml

For analyses focusing on associations between socioeconomic deprivation and hospitalisation or uptake, we first constructed socioeconomic deprivation groups for IMD using quintiles of their score for LSOAs in England. In 2015, 45% of the population in Merseyside lived in the most deprived quintile [22]. Association between socioeconomic status and IAI hospitalisations were assessed by fitting a negative binomial regression model (due to over-dispersion) offset by resident population, adjusting for age group, sex and year-by-year variability in influenza incidence by including fiscal year. To examine the association between age group and deprivation an interaction term was also included. We also stratified by age group and incidence rate ratios (IRR) were calculated along with 95% confidence intervals.

To estimate the association between socioeconomic deprivation and influenza vaccine uptake, uptake data for each LSOA were extracted at GP practice level. We used the LSOA level GP registered population, weighting vaccine uptake by proportion of population served by each GP practice, to create synthetic estimates of vaccine uptake at LSOA level for the 2014/15 and 2015/16 seasons. Vaccine uptake levels were classified into three categories in accordance with the national targets set by NHS England, DH and PHE [27]:

- 24-59 month olds (target: 40%)
- 65+ year olds (target: 75%)
- At risk groups under 65 years of age (target: 55%)

The association between socioeconomic deprivation and vaccine uptake was assessed using fractional logit regression and robust standard errors [28]. The dependent variable was vaccine uptake, included as a proportion, and the independent variable was IMD quintile. The model was adjusted for fiscal year (April to March) and, because the unit of analysis was LSOA, the model was weighted using LSOA population size. Three separate models were constructed for vaccine uptake in 24-59 month olds, 65+ year olds, and at-risk groups under 65 years of age. The models enabled the calculation of odds ratios (ORs) and robust 95% confidence intervals.

For peer review only - http://bmjopen.bmj.com/site/about/guidelines.xhtml

190 Demographic characteristics were compared between those with and without clinical risk factors for severe IAI. Continuous variables were tested by Student's t-test, or Wilcoxon rank-sum test if not normally distributed, and χ^2-test or Fisher's exact test were used for categorical variables.

193 All data processing and statistical analyses were conducted using R v. 3.2.3 [29].

Patient and public involvement

195 Through our institute's public involvement panel (PIP) the study team has had an ongoing dialogue on influenza vaccine research. This research project was generated from these discussions as PIP members identified that reducing inequalities in influenza vaccine uptake should be a policy priority. The panel have considered and commented on the aims and objectives, and contributed to the development of this study. The findings from this study have be shared with organisations involved in data collection, patient contact, and the PPI panel. Investigators have and will continue to present these findings at regional and national events and to participating NHS organisations (including lay membership), public health departments and government agencies.

Results

Hospitalisations in Merseyside

205 Between April 2004 and March 2016, there were 89,058 hospitalisation events coded as influenza or IAI in Merseyside (mean *per* year: 7,421.5; 4.9 per 1,000 population), of which 43,761 (49.1%) occurred among male patients (supplementary S1-Table 2). The highest number of hospitalisations occurred in the 2006/07 season (n=9,247; 6.2 per 1,000 population), and the lowest in 2011/12 (5,231; 3.5 per 1,000 population). Hospitalisations showed a clear seasonal pattern, peaking in December and January (Figure 1). Most hospitalisations (n=29,877; 33.5%) corresponded to patients aged 65 years and over, or children under 24 months of age (n=21,420; 24%). Over the study period, the mean population rate of hospitalisation was highest in children aged under 24 months of age (51.3 per 1,000) and 24-59 months of age (15.4 per 1,000).

214 During the 2009/10 influenza season, when the H1N1 pandemic occurred, the proportion of

For peer review only - http://bmjopen.bmj.com/site/about/guidelines.xhtml

hospitalisations corresponding to patients 65+ year olds was at its lowest (Figure 1; supplementary S1-Table 2), reflecting the shift towards younger adults observed during the H1N1 pandemic. Forty-eight percent (n=42,712) of hospitalisation had an ICD-10 clinical code that indicated a comorbidity. The two most common comorbidities were heart disease (n=19,293; 45%) and chronic respiratory conditions (n=15,755; 37%). The proportion of hospitalisations with comorbidities for complicated influenza increased with age, and was slightly greater in males (n=22,444; 53%).

Association between hospitalisation rates and socioeconomic deprivation

Over half of the hospitalisations (n=47,008; 53%) occurred among those living in the most socioeconomically deprived quintile (Table 1). Overall, the proportion of hospitalisations for IAI with a comorbidity was lower in the most deprived quintile (47%: n=22167/47008; p=0.01) compared to the least deprived (49%: n=2672/5454) (Table 1). In respect to age group, a similar proportion of children aged <24 months (range: 6-7%) and adults 65+ years of age (range 79-81%) had comorbidities across the deprivation strata (Table 1). However, among children aged 5-14 years and among 15-39 year olds and 40-64 years olds the proportion of IAI hospitalisations that had a comorbidity was highest in the most deprived quintiles (Table 1). Below the age of 5 years there was little difference in hospitalisation rates between the deprivation quintiles (Figure 2); for example, the mean rate of IAI hospitalisations over the study period was 26.5 per 1,000 population in the most deprived quintile and 31.4 per 1,000 population in the least deprived. For children aged 5-14 years the risk of IAI hospitalisation was 1.2 times among those from the most deprived compare to the least deprived (IRR=1.23: 95% CI; 1.04-1.47; p=0.019). In those aged 15-39 years the risk of hospitalisation was over 2 times higher (IRR=2.08: 95% CI; 1.76-2.45; p<0.001) among residents in the most deprived areas in comparison to those in the least deprived quintile; in adults aged 40-64 years it was 2.6 times higher (IRR=2.65: 95% CI; 2.35-2.99; p<0.001) and 1.9 times higher in 65+ year olds (IRR=1.90: 95% CI; 1.73-2.10; p<0.001). When including an interaction term for the association between IMD quintile and age group, socioeconomic deprivation was strongly associated with an increased rate of hospitalisation in the 15-39, 40-64 and 65+ year age group. Comparing the least

For peer review only - http://bmjopen.bmj.com/site/about/guidelines.xhtml

For peer review only

deprived IMD quintile with the most deprived and 40-64 year olds with <24 month olds, risk of hospitalisation was nearly 2.4 times higher (IRR= 2.39: 95% CI 1.94-2.95; p<0.001) (supplementary S1-Table 3). Spatially age and sex-standardised Incidence ratios (SIRs) for IAI hospitalisations also show a visual association with deprivation over time, with higher rates being consistently observed in the most deprived areas (Figure 3).

Table 1. Population of Merseyside admitted to hospital with IAI by national IMD quintile between 2004/05 and 2015/16 (N=89058).

	National Index of Multiple Derivation quintile										Total	
	5 (least deprived) (N=5454)		4 (N=10567)		3 (N=12189)		2 (N=13840)		1 (most deprived) (N=47008)			
**Sex**	n	%	n	%	n	%	n	%	n	%	n	%
Female	2773	51	5394	51	6192	51	7121	51	23809	51	45289	51
Male	2681	49	5173	49	5997	49	6719	49	23197	49	43767	49
Unknown	0	0	0	0	0	0	0	0	2	0	2	0
**Age group**												
<24m	1205	22	2344	22	2898	24	3195	23	11779	25	21421	24
24-59m	607	11	1115	11	1290	11	1444	10	4917	10	9373	11
5-14y	385	7	679	6	634	5	801	6	2629	6	5128	6
15-39y	343	6	769	7	1079	9	1290	9	5399	11	8880	10
40-64y	742	14	1517	14	1883	15	2131	15	8096	17	14369	16
65+	2172	40	4143	39	4405	36	4979	36	14188	30	29887	34
**Comorbidity**												
No	2782	51	5297	50	6389	52	7037	51	24841	53	46346	52
Yes	2672	49	5270	50	5800	48	6803	49	22167	47	42712	48
**Comorbidity by age group**†												
<24m	73	6	148	6	187	6	202	6	841	7	1451	7
24-59m	112	18	216	19	231	18	302	21	1126	23	1987	21
5-14y	141	37	268	39	231	36	349	44	1126	43	2115	41
15-39y	145	42	361	47	479	44	625	48	2598	48	4208	47
40-64y	439	59	961	63	1183	63	1349	63	5292	65	9224	64
65+	1762	81	3316	80	3489	79	3976	80	11184	79	23727	79

† Denominator for percentages is the total number of hospitalisations in each age group for each deprivation quintile. m: months; y: years.

Influenza Vaccine Uptake

Vaccine uptake among children aged 2-4 years for the 2014/15 and 2015/16 season did not attain the DH target of 40% in most of Merseyside (Figure 4a). In 2015/16, 916/989 (93%) LSOAs did not

For peer review only - http://bmjopen.bmj.com/site/about/guidelines.xhtml

meet the 40% target. In 2015/16. Vaccine uptake among those aged 65+ in the 2015/16 season was below the 75% DH target, with 701/989 (71%) LSOA areas below the target (Figure 4b). Among those presenting risk factors for complicated influenza, vaccine uptake in Merseyside was also below the DH target of 55%, with 853/989 (86%) LSOA areas not meeting the target in 2015/16 (Figure 4c). Vaccine uptake was lowest in the most socioeconomically deprived quintiles (Figure 5). In the 2014/15 and 2015/16 seasons, the odds of influenza vaccine uptake in the most deprived quintile compared to the least deprived were 30% lower in the 24-59 months group (OR= 0.70: 95% CI; 0.66-0.74; p<0.001), 10% lower in the 65+ year olds (OR= 0.90: 95% CI; 0.88-0.92; p<0.001). In at risk groups there was no major difference, 3% lower in most deprived (OR=1.00: 95%; CI 0.98-1.04; p=0.66).

Discussion

This study shows variations in the number of hospitalisations for IAI in Merseyside, England, across the influenza seasons from 2004/5 to 2015/16, with a significant measurable difference by socioeconomic deprivation status in the adult population, which is in concordance with findings from other settings [8,30,31]. Hospitalised IAI rates among those aged 40-64 years living in the most deprived communities were more than double those of the same age group from the most affluent areas. Influenza vaccine uptake was below the national DH target among those aged <65 years presenting comorbidities, and while it was close to the 75% target for the >65 in 20014/15, most of Merseyside was below target in 2015/16.

In contrast to our findings among adults, IAI hospitalisation rates among children <5 years of age were similar across socioeconomic strata, and vaccination uptake remained consistently below the 40% DH target for both 2014/15 and 2015/16 seasons. It could be hypothesised that in childhood, in the absence of comorbidities that could exacerbate the disease, hospitalisation following exposure to the influenza virus is likely to be driven by factors other than social determinants of health. While there is robust evidence indicating that social and economic disadvantage is strongly associated with

For peer review only - http://bmjopen.bmj.com/site/about/guidelines.xhtml

the development of ill-health in childhood and in later life [32], our results suggest that hospitalisations for infectious respiratory diseases, such as influenza illness, are an equal threat to very young children regardless of their socioeconomic background, which is likely to be related to there being less of a differential in comorbidities across deprivation strata at a young age.

While data on vaccine uptake was not available for influenza seasons prior to 2014/15, hospitalisation rates have followed a consistent pattern throughout the study period, with an age-shift observed in the 2009/10 season, consistent with the H1N1 pandemic [33]. Consistent failure to attain the DH vaccination targets across all eligible groups warrant further investigations. Accessibility to vaccination, knowledge and perceptions of both vaccination and disease are critical aspects of explaining and tackling low uptake of influenza vaccination. The interaction and relative importance of these issues will vary between populations, and will differ between adults and children. There is evidence that individuals at high-risk of complicated influenza infection do not perceive themselves to be susceptible to influenza illness, and that they have concerns over adverse effects of vaccination [34–36], which suggest that providing knowledge and education about influenza disease to high-risk groups will be crucial for any interventions attempting to improve vaccine uptake. Our findings further highlight the need for informed targeted interventions addressing low vaccine uptake among children and individuals at risk of complicated influenza to ensure national vaccination targets are met in coming years. The lower vaccine uptake rates observed among the most socioeconomically deprived populations compared the most affluent ones suggest Interventions should prioritise areas where vaccine uptake is low and hospitalisation rates are high. Vaccination with the seasonal influenza vaccine can induce long-term cross-reactive antibodies that could have a protective effect in the event of an influenza pandemic [37,38]. Highlighting this added benefit of the vaccine could potentially have a positive influence in people's perception of influenza vaccine, and contribute to improve vaccine uptake.

For peer review only - http://bmjopen.bmj.com/site/about/guidelines.xhtml

The strongest association between IAI hospitalisation and deprivation was seen among those aged 40-64. This could be in great part explained by the fact that unhealthy behaviours (*e.g.* smoking, alcohol abuse) and environmental risk factors (e.g. poor living environment) are associated with lower socioeconomic status. These factors will contribute cumulatively to comorbidities over time peaking in middle to old age [39], and will, in turn, have a negative impact in on life expectancy, which is lower in deprived areas [40]. While, vaccine uptake in vaccine eligible 65+ year olds is significantly higher than in those aged 40-64 years, the association between IAI hospitalisation and deprivation is still clearly present, though slightly less pronounced. Interventions designed with the aim of improving the overall health and quality of life of the adults in the most socioeconomically deprived populations can potentially contribute to a reduction in IAI [41].

Limitations

Reliance on ICD-10 codes for the identification of IAI is an important limitation to our study. While we can assume that ICD-10 coding for confirmed influenza denotes laboratory confirmation, many hospitalised influenza cases will be unconfirmed by laboratory diagnostics and are likely to receive a symptom-based ICD-10 diagnosis code on discharge. Consequently, the true burden of influenza hospitalisations could be higher or lower than our estimations. The use of a seasonal usage ratio to help identify ICD-10 codes associated with IAI was identified *a-priori* through professional consultation but unvalidated against a gold standard diagnostic. However, the selection of ICD-10 codes used for this study was carefully assessed and informed by previous studies [2,24,25], in an attempt to minimize misclassification. Crucially for the research aims, variations in coding over time were not observed, nor are they likely to be influenced by the socioeconomic background of the patient, since we used data from the national health system. Therefore, we are reassured that the observed association and direction of the effect have not been affected by using ICD-10 coding.

Another important limitation identified in this study is the fact that information is recorded in several databases throughout the health system without common identifiers to enable the data

For peer review only - http://bmjopen.bmj.com/site/about/guidelines.xhtml

linkage of individuals. While hospitalisation data could be identified to LSOA level through HES, vaccine uptake could only be obtained at GP practice level through ImmForm, and was synthetically estimated at LSOA level. Moreover, none of these databases are linked to laboratory information. While the correlation with socioeconomic deprivation was demonstrated for both outcomes, and is apparent in our graphic representations, this limitation prevented the development of more complex spatio-temporal Bayesian hierarchical models. In order to evaluate interventions aimed at improving vaccine uptake and reducing the burden of hospitalisations due to IAI, surveillance data on disease incidence, hospitalisations, and vaccine uptake should be recorded in a systematic and consistent manner over time to facilitate comparison before and after the intervention was implemented. A surveillance system facilitating record matching between the various existing databases and the incorporation of laboratory data would facilitate recording disease trends and the identification of any changes in disease epidemiology, naturally or as a consequence of a public health intervention.

Finally, while our study has been able to demonstrate the association between socioeconomic deprivation and IAI hospitalisations, and with lower vaccine uptake, the results cannot elucidate the underlying causes for this observation. Comorbidities exacerbate respiratory disease[6], and socially-deprived areas tend to have more limited access to social resources, resulting in poorer health outcomes [42]. Therefore, designing effective vaccine policy and public health interventions that translate outcomes to health equity is critical; and requires improved understanding of the healthcare system engagement that underpins lower influenza vaccine uptake [43].

Declarations

Ethical approval: The data accessed was aggregated and anonymised and therefore as specified by the University of Liverpool ethics guidance and the *'NHS Health Research Authority Research Do I need NHS REC approval Tool'* (http://www.hra-decisiontools.org.uk/research/) this study did not require ethical approval.

For peer review only - http://bmjopen.bmj.com/site/about/guidelines.xhtml

Availability of data and material: The data that support the findings of this study are available from a Public Health England but restrictions apply to the availability of these data as they are not publicly available. Aggregated data may be available from the authors/ Public Health England upon reasonable request and with permission of Public Health England.

Potential competing interests: NF and DH were in receipt of research grant support on the topic of rotavirus vaccines from GlaxoSmithKline (GSK) Biologicals. DH is in receipt of research grant support on the topic of rotavirus vaccines from Sanofi Pasteur, and Merck & Co., Inc. (Kenilworth, NJ USA) after the closure of Sanofi Pasteur-MSD in December 2016.

Funding: This study was supported by a grant from NIHR Research Capacity Funding via Liverpool NHS clinical commissioning group.

Authors' contributions: AIP, DH, PC and NF conceived of and designed the study. DH acquired and analysed the data. All authors interpreted the data; DH and AIP wrote the first draft of the report; and all authors reviewed the draft and final manuscript.

Acknowledgements: The authors acknowledge the contribution of Gabbie Marr and Ed Gaynor at Liverpool NHS clinical commissioning group and Emer Coffey from Public Health, Liverpool City Council.

For peer review only

For peer review only - http://bmjopen.bmj.com/site/about/guidelines.xhtml

References

1. Pitman RJ, Melegaro A, Gelb D, Siddiqui MR, Gay NJ, Edmunds WJ. Assessing the burden of influenza and other respiratory infections in England and Wales. J Infect. 2007;54: 530–538. doi:10.1016/j.jinf.2006.09.017

2. Cromer D, van Hoek AJ, Jit M, Edmunds WJ, Fleming D, Miller E. The burden of influenza in England by age and clinical risk group: A statistical analysis to inform vaccine policy. J Infect. 2014;68: 363–371. doi:10.1016/j.jinf.2013.11.013

3. Fleming DM, Taylor RJ, Haguinet F, Schuck-Paim C, Logie J, Webb DJ, et al. Influenza-attributable burden in United Kingdom primary care. Epidemiol Infect. 2015; 1–11. doi:10.1017/S0950268815001119

4. Meier CR, Napalkov PN, Wegmüller Y, Jefferson T, Jick H. Population-based study on incidence, risk factors, clinical complications and drug utilisation associated with influenza in the United Kingdom. Eur J Clin Microbiol Infect Dis Off Publ Eur Soc Clin Microbiol. 2000;19: 834–842.

5. Pockett RD, Watkins J, McEwan P, Meier G. Burden of Illness in UK Subjects with Reported Respiratory Infections Vaccinated or Unvaccinated against Influenza: A Retrospective Observational Study. PloS One. 2015;10: e0134928. doi:10.1371/journal.pone.0134928

6. Glezen WP, Greenberg SB, Atmar RL, Piedra PA, Couch RB. Impact of respiratory virus infections on persons with chronic underlying conditions. JAMA. 2000;283: 499–505.

7. Balasegaram S, Ogilvie F, Glasswell A, Anderson C, Cleary V, Turbitt D, et al. Patterns of early transmission of pandemic influenza in London - link with deprivation. Influenza Other Respir Viruses. 2012;6: e35-41. doi:10.1111/j.1750-2659.2011.00327.x

8. Hawker JI, Olowokure B, Sufi F, Weinberg J, Gill N, Wilson RC. Social deprivation and hospital admission for respiratory infection: an ecological study. Respir Med. 2003;97: 1219–1224.

9. Rutter PD, Mytton OT, Mak M, Donaldson LJ. Socio-economic disparities in mortality due to pandemic influenza in England. Int J Public Health. 2012;57: 745–750. doi:10.1007/s00038-012-0337-1

10. Joint Committee on Vaccination and Immunisation. JCVI statement on the routine annual influenza vaccination programme - extension of the programme to children [Internet]. Department of Health; 2013 Jul pp. 1–6. Available: https://www.gov.uk/government/publications/jcvi-statement-on-the-routine-annual-influenza-vaccination-programme

11. Public Health England. Seasonal influenza vaccine uptake amongst GP Patients in England: winter season 2016 to 2017 [Internet]. London, England: Public Health England; 2017 Nov. Available: https://www.gov.uk/government/statistics/seasonal-flu-vaccine-uptake-in-gp-patients-in-england-winter-season-2016-to-2017

12. Public Health England. Seasonal flu vaccine uptake in children of primary school age: winter season 2016 to 2017 [Internet]. London, England: Public Health England; 2017 Nov. Available: https://www.gov.uk/government/statistics/seasonal-flu-vaccine-uptake-in-children-of-primary-school-age-winter-season-2016-to-2017

For peer review only - http://bmjopen.bmj.com/site/about/guidelines.xhtml

13. Public Health England. Who we are and what we do: Annual Plan 2015/16 [Internet]. London: Public Health England; 2015 Jul pp. 1–14. Available: https://www.gov.uk/government/uploads/system/uploads/attachment_data/file/452328/Annual_plan_2015-_Aug7-web.pdf

14. Hungerford D, Macpherson P, Farmer S, Ghebrehewet S, Seddon D, Vivancos R, et al. Effect of socioeconomic deprivation on uptake of measles, mumps and rubella vaccination in Liverpool, UK over 16 years: a longitudinal ecological study. Epidemiol Infect. 2015; 1–11. doi:10.1017/S0950268815002599

15. Green HK, Andrews N, Letley L, Sunderland A, White J, Pebody R. Phased introduction of a universal childhood influenza vaccination programme in England: population-level factors predicting variation in national uptake during the first year, 2013/14. Vaccine. 2015;33: 2620–2628. doi:10.1016/j.vaccine.2015.03.049

16. Spencer AM, Roberts SA, Brabin L, Patnick J, Verma A. Sociodemographic factors predicting mother's cervical screening and daughter's HPV vaccination uptake. J Epidemiol Community Health. 2014;68: 571–577. doi:10.1136/jech-2013-202629

17. Baguelin M, Flasche S, Camacho A, Demiris N, Miller E, Edmunds WJ. Assessing Optimal Target Populations for Influenza Vaccination Programmes: An Evidence Synthesis and Modelling Study. PLoS Med. 2013;10: e1001527. doi:10.1371/journal.pmed.1001527

18. Baguelin M, Hoek AJV, Jit M, Flasche S, White PJ, Edmunds WJ. Vaccination against pandemic influenza A/H1N1v in England: a real-time economic evaluation. Vaccine. 2010;28: 2370–2384. doi:10.1016/j.vaccine.2010.01.002

19. Hiscock R, Bauld L, Amos A, Platt S. Smoking and socioeconomic status in England: the rise of the never smoker and the disadvantaged smoker. J Public Health Oxf Engl. 2012;34: 390–396. doi:10.1093/pubmed/fds012

20. Newton JN, Briggs ADM, Murray CJL, Dicker D, Foreman KJ, Wang H, et al. Changes in health in England, with analysis by English regions and areas of deprivation, 1990-2013: a systematic analysis for the Global Burden of Disease Study 2013. Lancet Lond Engl. 2015;386: 2257–2274. doi:10.1016/S0140-6736(15)00195-6

21. Office for National Statistics. Lower Super Output Area Mid-Year Population Estimates [Internet]. 26 Oct 2016 [cited 5 Jul 2017]. Available: https://www.ons.gov.uk/peoplepopulationandcommunity/populationandmigration/populationestimates/datasets/lowersuperoutputareamidyearpopulationestimates

22. Department for Communities and Local Government. English indices of deprivation 2015 [Internet]. London, England: Department for Communities and Local Government; 2015 Sep. Available: https://www.gov.uk/government/statistics/english-indices-of-deprivation-2015

23. Atkins K, Hoek AJ van, Watson C, Baguelin M, Choga L, Patel A, et al. Seasonal influenza vaccination delivery through community pharmacists in England: evaluation of the London pilot. BMJ Open. 2016;6: e009739. doi:10.1136/bmjopen-2015-009739

24. Moore HC, Lehmann D, Klerk N de, Smith DW, Richmond PC, Keil AD, et al. How Accurate Are International Classification of Diseases-10 Diagnosis Codes in Detecting Influenza and Pertussis Hospitalizations in Children? J Pediatr Infect Dis Soc. 2014;3: 255–260. doi:10.1093/jpids/pit036

For peer review only - http://bmjopen.bmj.com/site/about/guidelines.xhtml

25. Moore K, Black J, Rowe S, Franklin L. Syndromic surveillance for influenza in two hospital emergency departments. Relationships between ICD-10 codes and notified cases, before and during a pandemic. BMC Public Health. 2011;11: 338. doi:10.1186/1471-2458-11-338

26. Buda S, Tolksdorf K, Schuler E, Kuhlen R, Haas W. Establishing an ICD-10 code based SARI-surveillance in Germany - description of the system and first results from five recent influenza seasons. BMC Public Health. 2017;17: 612. doi:10.1186/s12889-017-4515-1

27. Public Health England. Flu plan: winter 2016 to 2017 [Internet]. London, England: Public Health England; 2017 May. Available: https://www.gov.uk/government/publications/flu-plan-winter-2016-to-2017

28. Papke LE, Wooldridge JM. Econometric methods for fractional response variables with an application to 401(k) plan participation rates. J Appl Econom. 1996;11: 619–632. doi:10.1002/(SICI)1099-1255(199611)11:6<619::AID-JAE418>3.0.CO;2-1

29. R Core Team. R: A Language and Environment for Statistical Computing [Internet]. Vienna, Austria: R Foundation for Statistical Computing; 2016. Available: http://www.R-project.org

30. Charland KM, Brownstein JS, Verma A, Brien S, Buckeridge DL. Socio-economic disparities in the burden of seasonal influenza: the effect of social and material deprivation on rates of influenza infection. PloS One PLoS ONE. 2011;6, 6: e17207–e17207. doi:10.1371/journal.pone.0017207, 10.1371/journal.pone.0017207

31. Hyder A, Leung B. Social deprivation and burden of influenza: Testing hypotheses and gaining insights from a simulation model for the spread of influenza. Epidemics. 2015;11: 71–79. doi:10.1016/j.epidem.2015.03.004

32. Wickham S, Anwar E, Barr B, Law C, Taylor-Robinson D. Poverty and child health in the UK: using evidence for action. Arch Dis Child. 2016;101: 759. doi:10.1136/archdischild-2014-306746

33. Falagas ME, Koletsi PK, Baskouta E, Rafailidis PI, Dimopoulos G, Karageorgopoulos DE. Pandemic A(H1N1) 2009 influenza: review of the Southern Hemisphere experience. Epidemiol Infect. 2011;139: 27–40. doi:10.1017/S0950268810002037

34. Cornford CS, Morgan M. Elderly people’s beliefs about influenza vaccination. Br J Gen Pract J R Coll Gen Pract. 1999;49: 281–284.

35. Blank PR, Schwenkglenks M, Szucs TD. Disparities in influenza vaccination coverage rates by target group in five European countries: trends over seven consecutive seasons. Infection. 2009;37: 390–400. doi:10.1007/s15010-009-8467-y

36. Santos AJ, Kislaya I, Machado A, Nunes B. Beliefs and attitudes towards the influenza vaccine in high-risk individuals. Epidemiol Infect. 2017;145: 1786–1796. doi:10.1017/S0950268817000814

37. McCullers JA, Van De Velde L-A, Allison KJ, Branum KC, Webby RJ, Flynn PM. Recipients of vaccine against the 1976 “swine flu” have enhanced neutralization responses to the 2009 novel H1N1 influenza virus. Clin Infect Dis Off Publ Infect Dis Soc Am. 2010;50: 1487–1492. doi:10.1086/652441

For peer review only

For peer review only - http://bmjopen.bmj.com/site/about/guidelines.xhtml

38. Wei C-J, Boyington JC, Dai K, Houser KV, Pearce MB, Kong W-P, et al. Cross-neutralization of 1918 and 2009 influenza viruses: role of glycans in viral evolution and vaccine design. Sci Transl Med. 2010;2: 24ra21. doi:10.1126/scitranslmed.3000799

39. Bonnie RJ, Stratton K, Kwan LY, Products C on the PHI of R the MA for PT, Practice B on PH and PH, Medicine I of. The Effects of Tobacco Use on Health [Internet]. National Academies Press (US); 2015. Available: https://www.ncbi.nlm.nih.gov/books/NBK310413/

40. Health state life expectancies by Index of Multiple Deprivation (IMD) - Office for National Statistics [Internet]. [cited 22 Jan 2018]. Available: https://www.ons.gov.uk/peoplepopulationandcommunity/healthandsocialcare/healthinequalities/bulletins/healthstatelifeexpectanciesbyindexofmultipledeprivationimd/england2013to2015

41. Lawson KD, Mercer SW, Wyke S, Grieve E, Guthrie B, Watt GC, et al. Double trouble: the impact of multimorbidity and deprivation on preference-weighted health related quality of life a cross sectional analysis of the Scottish Health Survey. Int J Equity Health. 2013;12: 67. doi:10.1186/1475-9276-12-67

42. Zhang X, Cook PA, Lisboa PJ, Jarman IH, Bellis MA. The effects of deprivation and relative deprivation on self-reported morbidity in England: an area-level ecological study. Int J Health Geogr. 2013;12: 5. doi:10.1186/1476-072X-12-5

43. Nagata JM, Hernández-Ramos I, Kurup AS, Albrecht D, Vivas-Torrealba C, Franco-Paredes C. Social determinants of health and seasonal influenza vaccination in adults ≥65 years: a systematic review of qualitative and quantitative data. BMC Public Health. 2013;13: 388. doi:10.1186/1471-2458-13-388

For peer review only

For peer review only - http://bmjopen.bmj.com/site/about/guidelines.xhtml

Figure 1. Number of hospitalisations for IAI by Merseyside residents, 5 week moving average 2004/05-2015/16, by age-groups. m: months; y: years

Figure 2. Incidence rate ratios of IAI hospitalisation rate for Index of Multiple Deprivation quintiles 1 (most deprived)-4 compared to a reference group of the least deprived quintile (5) by age group

Rate ratios for IAI hospitalised incidence rates were estimated with negative binomial regression stratified by age group. Models were additionally adjusted for sex and fiscal year. m: months; y: years. * $p < 0.05$, ** $p < 0.01$, ***$p < 0.001$

Figure 3. Age and sex standardised incidence ratios for IAI hospitalisations for Merseyside residents by MSOA and pooled years, 2004/05 - 2015/16

Figure 4. Influenza vaccine uptake by LSOA 2014/15 and 2015/16 seasons in: a) 24-59 month olds (40% NHS England target); b) 65+ year olds (75% NHS England target); c) at risk groups aged 6 months to 64 years (55% NHS England target). Each block represents 1 of 989 LSOAs in Merseyside.

Figure 5. Odds ratios of vaccine uptake by Index of Multiple Deprivation quintiles 1 (most deprived)-4 compared to a reference group of the least deprived quintile (5) by age group, 2014/15-2015/16

Adjusted odds ratios for vaccine uptake were estimated with general linear models of the binomial family with a logit function, robust standard errors were used to calculate 95% confidence intervals. Models were additionally adjusted for fiscal year and weighted using the relevant age related population of each LSOA. m, months.

* $p < 0.05$, ** $p < 0.01$, ***$p < 0.001$

For peer review only - http://bmjopen.bmj.com/site/about/guidelines.xhtml

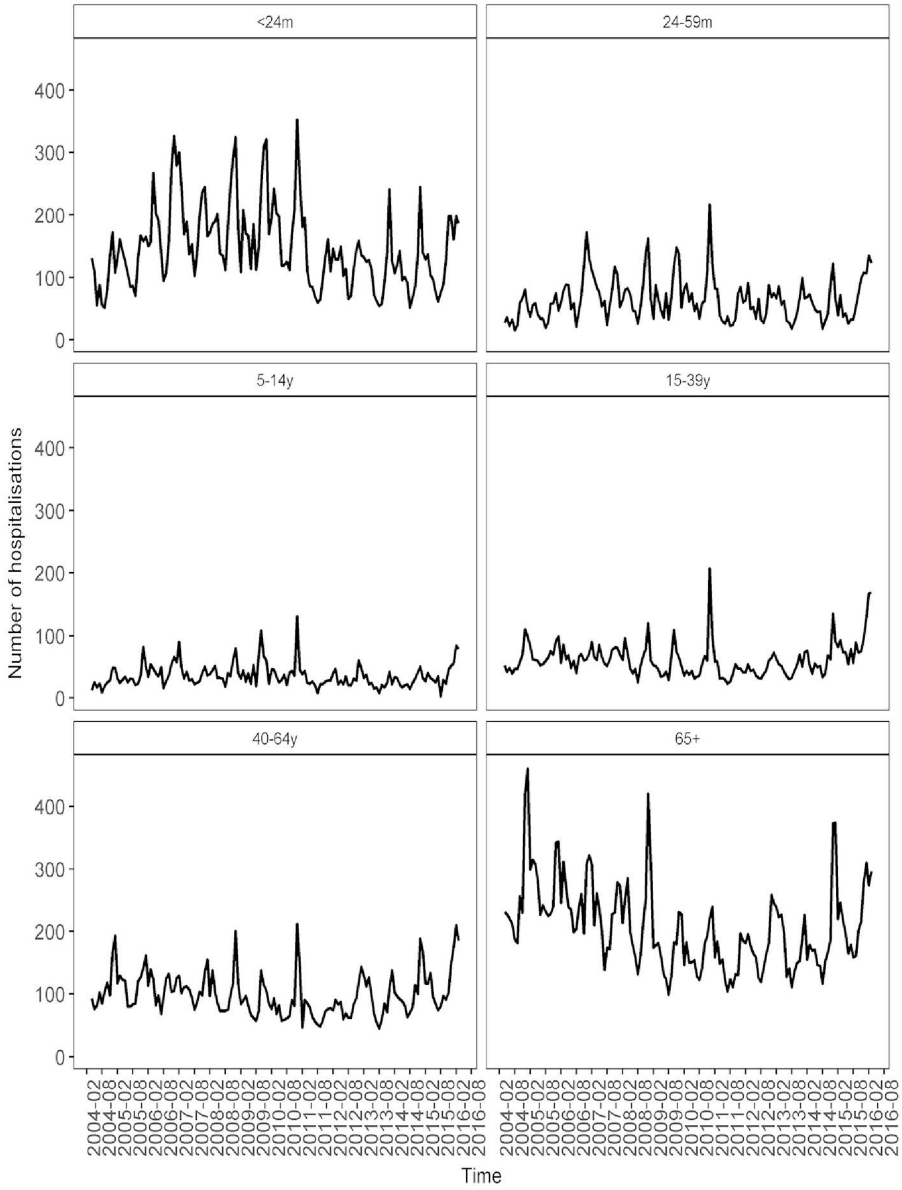

Figure 1. Number of hospitalisations for IAI by Merseyside residents, 5 week moving average 2004/05-2015/16, by age-groups

199x266mm (300 x 300 DPI)

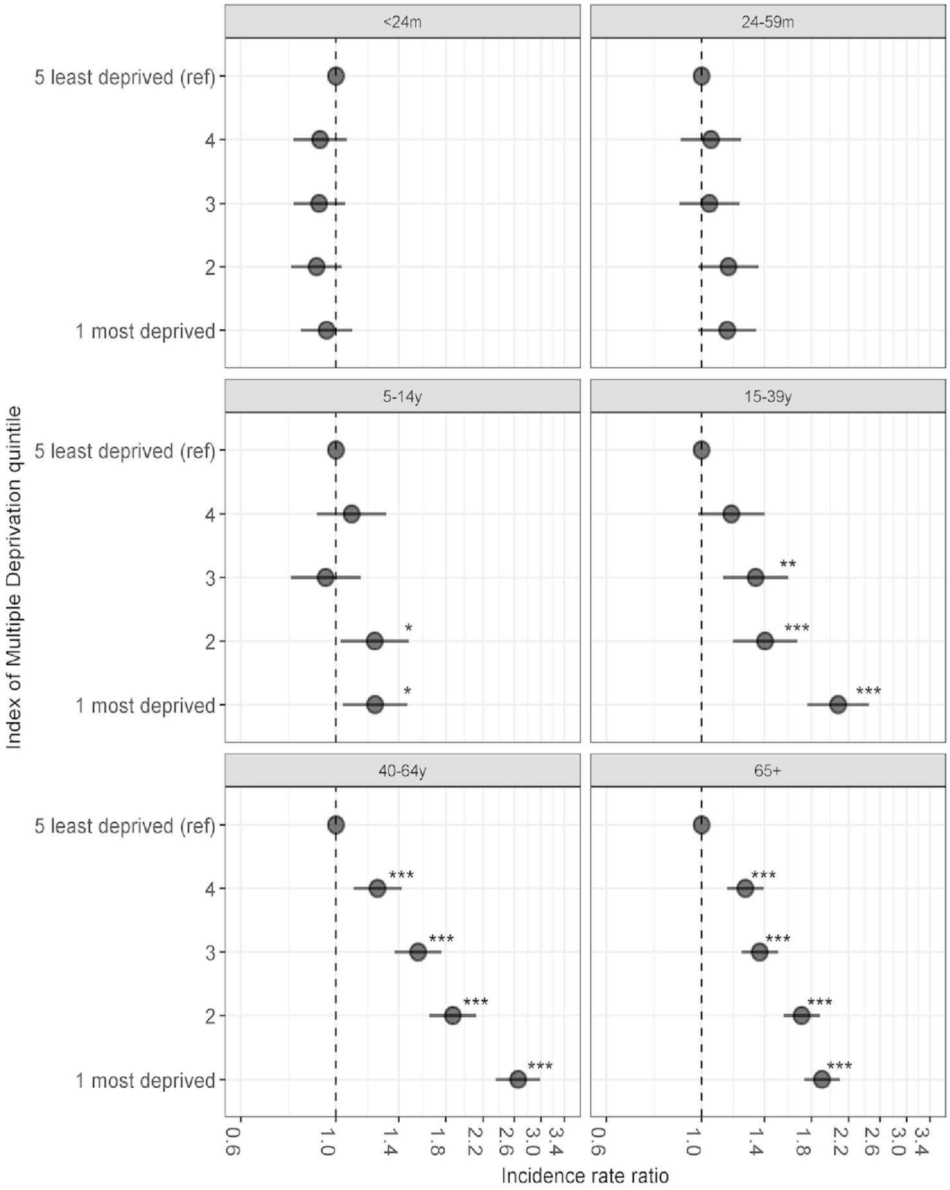

Figure 2. Incidence rate ratios of IAI hospitalisation rate for Index of Multiple Deprivation quintiles 1 (most deprived)-4 compared to a reference group of the least deprived quintile (5) by age group

180x224mm (300 x 300 DPI)

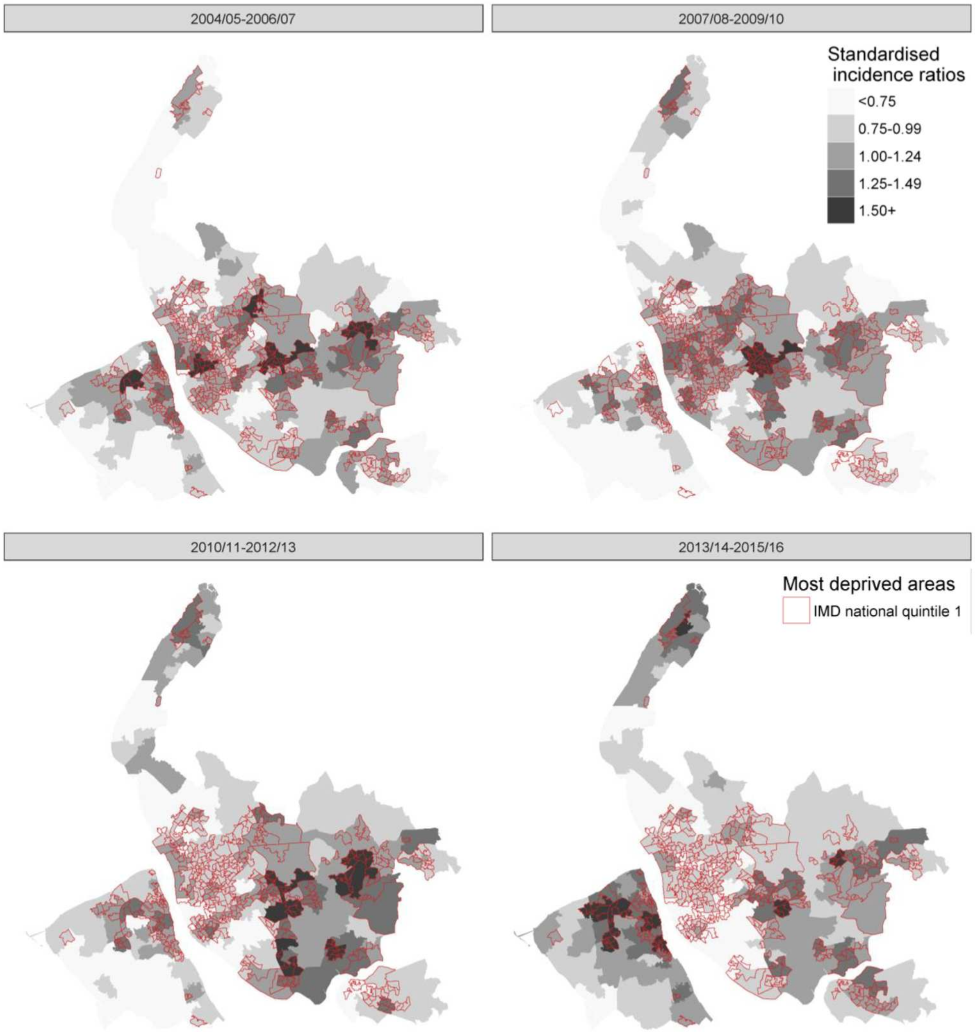

Figure 3. Age and sex standardised incidence ratios for IAI hospitalisations for Merseyside residents by MSOA and pooled years, 2004/05 - 2015/16

174x183mm (300 x 300 DPI)

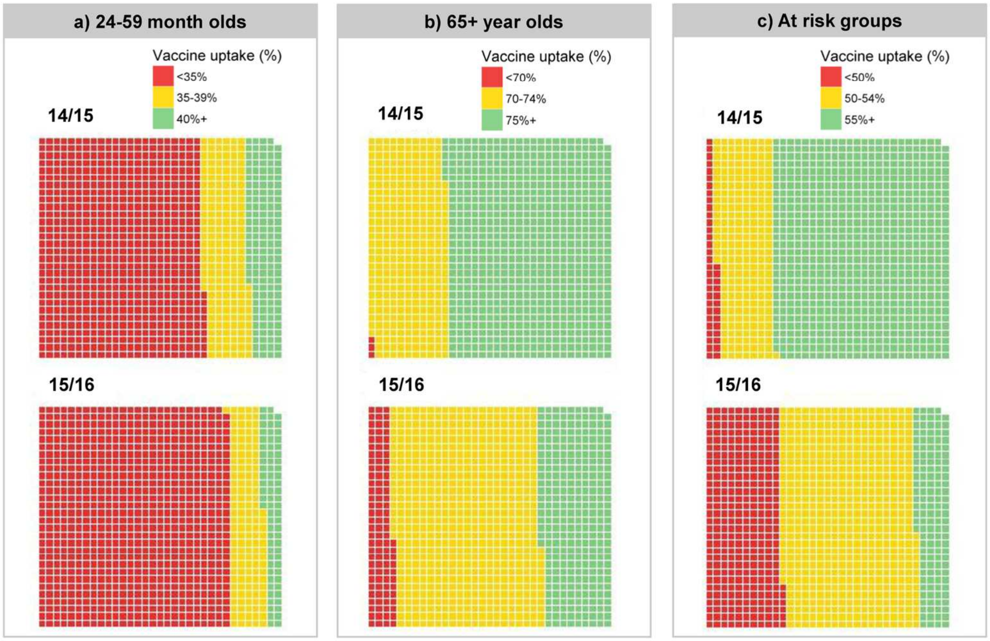

Figure 4. Influenza vaccine uptake by LSOA 2014/15 and 2015/16 seasons in: a) 24-59 month olds (40% NHS England target); b) 65+ year olds (75% NHS England target); c) at risk groups aged 6 months to 64 years (55% NHS England target).

151x100mm (300 x 300 DPI)

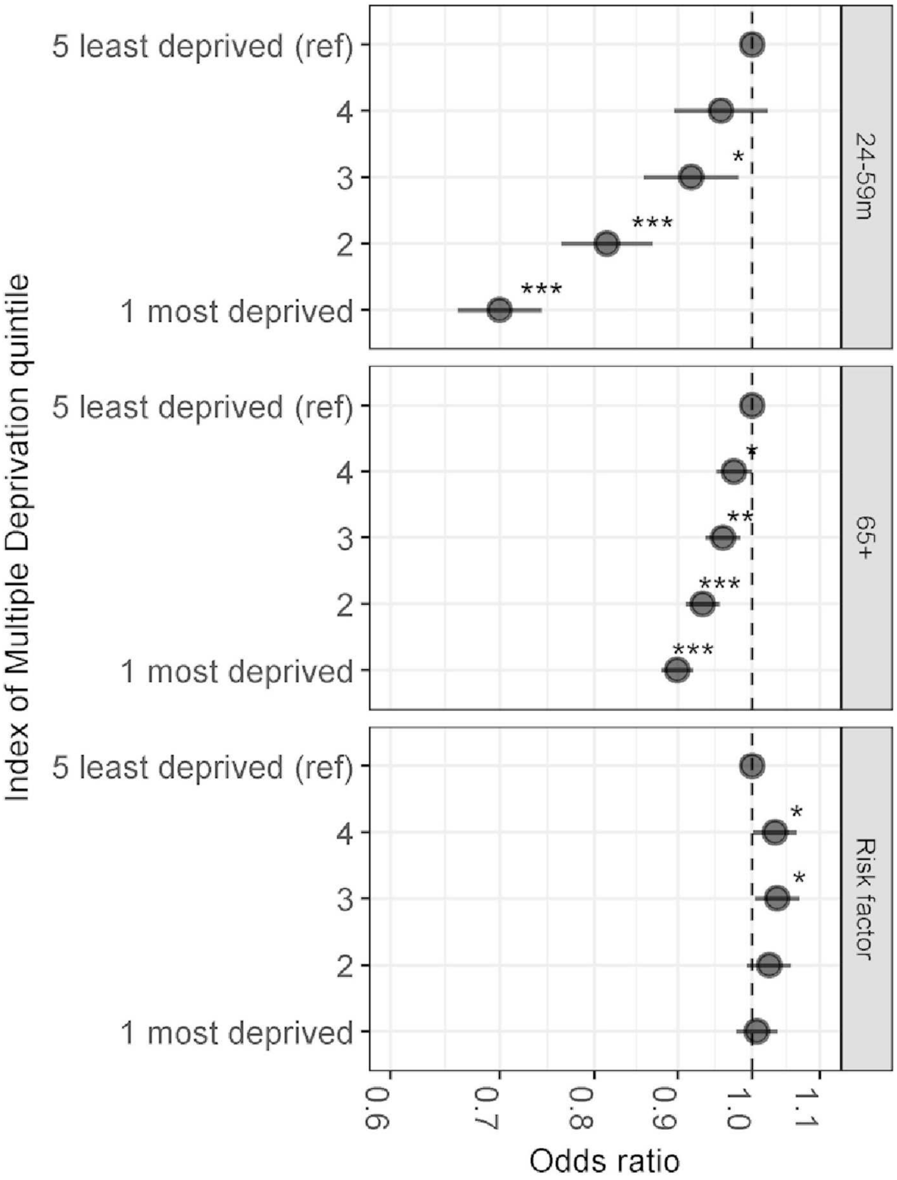

Figure 5. Odds ratios of vaccine uptake by Index of Multiple Deprivation quintiles 1 (most deprived)-4 compared to a reference group of the least deprived quintile (5) by age group, 2014/15-2015/16

129x168mm (300 x 300 DPI)

S1-Table 1. ICD-10 codes used to identify comorbidities adapted from Cromer et al. 2014

Condition	ICD-10 codes used
**Chronic respiratory disease**	J4, J6, J7, J8, Q30, J31, Q32, Q33Q34, Q35, Q36, Q37
**Chronic heart disease**	I05, I06, I07, I08, I09, I11, I12, I13, I20, I21, I22, I25, I27, I28, I3, I40, I41, I42, I43, I44, I45, I47, I48, I49, I5, I6, Q2
**Chronic kidney disease**	N0, N11, N12, N14, N15, N16, N18, N19, N25, Q60, Q61
**Chronic liver disease**	K7, P78.8, Q44, B18
**Chronic neurological disease**	G1, G2, G3, G4, G5, G6, G7, G8, G9
**Diabetes**	E10, E11, E12, E13, E14, E24, G59.0, G63.2, G73.0, G99.0, N08.3, O24, P70.0, P70.1, P70.2
**Immunosuppression**	All C-codes, D37, D38, D39, D4, B20, B21, B22, B23, B24, Z94, Z85, Z94.8, D56.1, D57.8, D57.0, D57.D61, D70, D71, D72, D73, D76, D80, D81, D82, D83, D84, K90.0
**Other including: asplenia or dysfunction of the spleen, cochlear implants and cerebrospinal fluid leaks**	D73, D56.1, D57.8, D57.0, D57.1, K90.0, Z96.1, G96.0

For peer review only

S1-Table 2. Demographics of Merseyside residents admitted to hospital with IAI (ICD-10: J09, J10, J128, J129, J11, J22, J069) by financial year, 2004/05 to 2015/16 (N=89,058)

	04/05 (N=7457)		05/06 (N=8018)		06/07 (N=9248)		07/08 (N=8175)		08/09 (N=8269)		09/10 (N=7559)		10/11 (N=7640)		11/12 (N=5231)		12/13 (N=6399)		13/14 (N=5883)		14/15 (N=6985)		15/16 (N=8194)	
Sex	**n**	**%**	**n**	**%**	**n**	**%**	**n**	**%**	**n**	**%**	**n**	**%**	**n**	**%**	**n**	**%**	**n**	**%**	**n**	**%**	**n**	**%**	**n**	**%**
Female	3900	52	4173	52	4572	49	4069	50	4171	50	3759	50	3777	49	2633	50	3267	51	3048	52	3730	53	4190	51
Male	3556	48	3845	48	4675	51	4106	50	4098	50	3800	50	3863	51	2598	50	3132	49	2835	48	3255	47	4004	49
Unknown	1	0	0	0	1	0	0	0	0	0	0	0	0	0	0	0	0	0	0	0	0	0	0	0
**Age group**																								
<24m	1281	17	1544	19	2583	28	2089	26	2310	28	2376	31	2235	29	1250	24	1441	23	1315	22	1425	20	1572	19
24-59m	510	7	597	7	1057	11	857	10	908	11	954	13	1013	13	589	11	692	11	643	11	680	10	873	11
5-14y	309	4	449	6	565	6	412	5	492	6	575	8	523	7	313	6	383	6	277	5	340	5	490	6
15-39y	752	10	825	10	761	8	844	10	708	9	628	8	785	10	463	9	581	9	605	10	797	11	1131	14
40-64y	1363	18	1376	17	1290	14	1312	16	1186	14	1049	14	1057	14	829	16	1104	17	1053	18	1277	18	1473	18
65+	3242	43	3227	40	2992	32	2661	33	2665	32	1977	26	2027	27	1787	34	2198	34	1990	34	2466	35	2655	32
**National IMD quintile**																								
5 (least deprived)	410	5	468	6	511	6	449	5	497	6	400	5	452	6	348	7	427	7	384	7	514	7	594	7
4	855	11	946	12	1005	11	959	12	914	11	831	11	862	11	654	13	837	13	764	13	880	13	1060	13
3	983	13	1107	14	1255	14	1038	13	1107	13	986	13	1007	13	762	15	996	16	856	15	998	14	1094	13
2	1151	15	1250	16	1445	16	1193	15	1292	16	1096	14	1096	14	818	16	1013	16	940	16	1149	16	1397	17
1 (most deprived)	4058	54	4247	53	5032	54	4536	55	4459	54	4246	56	4223	55	2649	51	3126	49	2939	50	3444	49	4049	49
**Comorbidity**																								
**No**	3617	49	4109	51	5442	59	4709	58	4528	55	4357	58	4360	57	2553	49	3046	48	2757	47	3073	44	3795	46
**Yes**	3840	51	3909	49	3806	41	3466	42	3741	45	3202	42	3280	43	2678	51	3353	52	3126	53	3912	56	4399	54

IMD: Index of Multiple Deprivation; m: months; y: years

For peer review only - http://bmjopen.bmj.com/site/about/guidelines.xhtml

S1-Table 3. Interaction between socioeconomic deprivation (IMD quintile) and age group on the rate of ILI hospitalisation in Merseyside, UK

	Mean yearly rate per 1,000 population	IRR	Full model with interaction: IRR	Standard error	P value
**Main effects**					
National IMD quintile					
5 (least deprived)	3.46	Reference	Reference		
4	3.98	1.13 (1.05-1.21)	1.00 (0.86-1.16)	0.077	0.984
3	4.26	1.18 (1.10-1.27)	0.99 (0.85-1.15)	0.076	0.864
2	4.82	1.37 (1.27-1.47)	1.04 (0.90-1.21)	0.076	0.581
1 (most deprived)	5.84	1.61 (1.50-1.72)	1.1 (0.95-1.27)	0.074	0.19
Age group					
<24m (reference)	51.3	Reference	Reference		
24-59m	15.4	0.30 (0.28-0.32)	0.29 (0.24-0.34)	0.084	<0.001
5-14y	2.49	0.05 (0.04-0.05)	0.05 (0.04-0.05)	0.090	<0.001
15-39y	1.50	0.03 (0.02-0.03)	0.02 (0.02-0.02)	0.091	<0.001
40-64y	2.43	0.04 (0.04-0.04)	0.03 (0.02-0.03)	0.082	<0.001
65+	9.66	0.18 (0.16-0.19	0.13 (0.11-0.15)	0.077	<0.001
Year	4.94	0.99 (0.98-1.00)	0.99 (0.98-1.00)	0.003	<0.001
Sex					
Male	5.01	Reference	Reference		
Female	4.88	0.98 (0.94-1.02)	0.98 (0.94-1.02)	0.020	0.263
**Interaction effects (IMD quintile interacted with age group)**					
IMD quintile 5 * <24m			reference		
IMD quintile 4 * 24-59m	-	-	1.05 (0.84-1.31)	0.114	0.663
IMD quintile 3 * 24-59m	-	-	1.06 (0.85-1.32)	0.113	0.628
IMD quintile 2* 24-59m	-	-	1.10 (0.89-1.38)	0.113	0.38
IMD quintile 1 * 24-59m	-	-	1.04 (0.84-1.29)	0.109	0.736
IMD quintile 4 * 5-14y	-	-	1.08 (0.85-1.37)	0.121	0.524
IMD quintile 3 * 5-14y	-	-	0.95 (0.75-1.21)	0.121	0.7
IMD quintile 2 * 5-14y	-	-	1.17 (0.93-1.48)	0.119	0.187
IMD quintile 1 * 5-14y	-	-	1.11 (0.88-1.39)	0.114	0.375
IMD quintile 4 * 15-39y	-	-	1.17 (0.92-1.48)	0.121	0.205
IMD quintile 3 * 15-39y	-	-	1.33 (1.06-1.68)	0.119	0.016
IMD quintile 2 * 15-39y	-	-	1.34 (1.06-1.69)	0.118	0.014
IMD quintile 1 * 15-39y	-	-	1.88 (1.50-2.35)	0.115	<0.001
IMD quintile 4 * 40-64y	-	-	1.25 (1.00-1.55)	0.112	0.05
IMD quintile 3 * 40-64y	-	-	1.57 (1.26-1.95)	0.111	<0.001
IMD quintile 2 * 40-64y	-	-	1.79 (1.44-2.22)	0.110	<0.001
IMD quintile 1 * 40-64y	-	-	2.39 (1.94-2.95)	0.108	<0.001
IMD quintile 4 * 65+	-	-	1.26 (1.03-1.56)	0.106	0.026
IMD quintile 3 * 65+	-	-	1.39 (1.13-1.71)	0.105	0.002
IMD quintile 2 * 65+	-	-	1.65 (1.34-2.02)	0.105	<0.001
IMD quintile 1 * 65+	-	-	1.75 (1.43-2.14)	0.103	<0.001

IMD: Index of Multiple deprivation; IRR: Incidence rate ratio; m: months; y: years

For peer review only

STROBE 2007 (v4) Statement—Checklist of items that should be included in reports of *cross-sectional studies*

Section/Topic	Item #	Recommendation	Reported on page #
**Title and abstract**	1	(*a*) Indicate the study's design with a commonly used term in the title or the abstract	Pg 1,2
		(*b*) Provide in the abstract an informative and balanced summary of what was done and what was found	Pg 2
**Introduction**			
Background/rationale	2	Explain the scientific background and rationale for the investigation being reported	Pg 4-5
Objectives	3	State specific objectives, including any prespecified hypotheses	Pg 5
**Methods**			
Study design	4	Present key elements of study design early in the paper	Pg 5-9
Setting	5	Describe the setting, locations, and relevant dates, including periods of recruitment, exposure, follow-up, and data collection	Pg 5,6
Participants	6	(*a*) Give the eligibility criteria, and the sources and methods of selection of participants	Pg 5-7
Variables	7	Clearly define all outcomes, exposures, predictors, potential confounders, and effect modifiers. Give diagnostic criteria, if applicable	Pg 6-9
Data sources/ measurement	8*	For each variable of interest, give sources of data and details of methods of assessment (measurement). Describe comparability of assessment methods if there is more than one group	Pg 6-8
Bias	9	Describe any efforts to address potential sources of bias	Pg 6-7
Study size	10	Explain how the study size was arrived at	NA
Quantitative variables	11	Explain how quantitative variables were handled in the analyses. If applicable, describe which groupings were chosen and why	Pg 8-9
Statistical methods	12	(*a*) Describe all statistical methods, including those used to control for confounding	Pg 8-10
		(*b*) Describe any methods used to examine subgroups and interactions	Pg 7-9
		(*c*) Explain how missing data were addressed	NA
		(*d*) If applicable, describe analytical methods taking account of sampling strategy	Pg 7-9
		(*e*) Describe any sensitivity analyses	Pg 6-7
**Results**			

For peer review only

Participants	13*	(a) Report numbers of individuals at each stage of study—eg numbers potentially eligible, examined for eligibility, confirmed eligible, included in the study, completing follow-up, and analysed	Pg 10, Table 1 and Supplementary Table 2
		(b) Give reasons for non-participation at each stage	NA
		(c) Consider use of a flow diagram	NA
Descriptive data	14*	(a) Give characteristics of study participants (eg demographic, clinical, social) and information on exposures and potential confounders	Pg 10,.11 12, Table 1 and Supplementary Table 2
		(b) Indicate number of participants with missing data for each variable of interest	NA
Outcome data	15*	Report numbers of outcome events or summary measures	Pg 10-12
Main results	16	(*a*) Give unadjusted estimates and, if applicable, confounder-adjusted estimates and their precision (eg, 95% confidence interval). Make clear which confounders were adjusted for and why they were included	Pg 11, 12 and 13 Figures 2 and 5
		(*b*) Report category boundaries when continuous variables were categorized	IMD
		(*c*) If relevant, consider translating estimates of relative risk into absolute risk for a meaningful time period	NA
Other analyses	17	Report other analyses done—eg analyses of subgroups and interactions, and sensitivity analyses	Pg 11-12. Supplementary table 3
**Discussion**			Pg 13-16
Key results	18	Summarise key results with reference to study objectives	Pg 13
Limitations	19	Discuss limitations of the study, taking into account sources of potential bias or imprecision. Discuss both direction and magnitude of any potential bias	Pg 15-16
Interpretation	20	Give a cautious overall interpretation of results considering objectives, limitations, multiplicity of analyses, results from similar studies, and other relevant evidence	Pg 13-16
Generalisability	21	Discuss the generalisability (external validity) of the study results	Pg 13-16
**Other information**			
Funding	22	Give the source of funding and the role of the funders for the present study and, if applicable, for the original study on which the present article is based	Pg17

*Give information separately for cases and controls in case-control studies and, if applicable, for exposed and unexposed groups in cohort and cross-sectional studies.

For peer review only

Note: An Explanation and Elaboration article discusses each checklist item and gives methodological background and published examples of transparent reporting. The STROBE checklist is best used in conjunction with this article (freely available on the Web sites of PLoS Medicine at http://www.plosmedicine.org/, Annals of Internal Medicine at http://www.annals.org/, and Epidemiology at http://www.epidem.com/). Information on the STROBE Initiative is available at www.strobe-statement.org.

For peer review only
